# Enhanced solute transport and steady mechanical stimulation in a novel dynamic perifusion bioreactor increase the efficiency of the *in vitro* culture of ovarian cortical tissue strips

**DOI:** 10.3389/fbioe.2024.1310696

**Published:** 2024-02-08

**Authors:** Gionata Fragomeni, Luigi De Napoli, Vincenza De Gregorio, Vincenzo Genovese, Vincenza Barbato, Giuseppe Serratore, Giuseppe Morrone, Angela Travaglione, Andrea Candela, Roberto Gualtieri, Riccardo Talevi, Gerardo Catapano

**Affiliations:** ^1^ Department of Medical and Surgical Sciences, Magna Graecia University, Catanzaro, Italy; ^2^ Department of Mechanical, Energy and Management Engineering, University of Calabria, Rende, Italy; ^3^ Department of Biology, University of Naples Federico II, Complesso Universitario di Monte S. Angelo, Naples, Italy

**Keywords:** bioreactor, dynamic culture, fluid dynamic stimulation, mechanical stimulation, ovarian tissue, oxygen transport

## Abstract

**Introduction:** We report the development and preliminary evaluation of a novel dynamic bioreactor to culture ovarian cortical tissue strips that leverages tissue response to enhanced oxygen transport and adequate mechanical stimulation. *In vitro* multistep ovarian tissue static culture followed by mature oocyte generation, fertilization, and embryo transfer promises to use the reserve of dormant follicles. Unfortunately, static *in vitro* culture of ovarian tissue does not promote development of primordial to secondary follicles or sustain follicle viability and thereby limits the number of obtainable mature oocytes. Enhancing oxygen transport to and exerting mechanical stimulation on ovarian tissue in a dynamic bioreactor may more closely mimic the physiological microenvironment and thus promote follicle activation, development, and viability.

**Materials and Methods:** The most transport-effective dynamic bioreactor design was modified using 3D models of medium and oxygen transport to maximize strip perifusion and apply tissue fluid dynamic shear stresses and direct compressive strains to elicit tissue response. Prototypes of the final bioreactor design were manufactured with materials of varying cytocompatibility and assessed by testing the effect of leachables on sperm motility. Effectiveness of the bioreactor culture was characterized against static controls by culturing fresh bovine ovarian tissue strips for 7 days at 4.8 × 10^−5^ m/s medium filtration flux in air at −15% maximal total compressive strain and by assessing follicle development, health, and viability.

**Results and Conclusions:** Culture in dynamic bioreactors promoted effective oxygen transport to tissues and stimulated tissues with strains and fluid dynamic shear stresses that, although non-uniform, significantly influenced tissue metabolism. Tissue strip culture in bioreactors made of cytocompatible polypropylene preserved follicle viability and promoted follicle development better than static culture, less so in bioreactors made of cytotoxic ABS-like resin.

## 1 Introduction

Replicating natural *folliculogenesis* *in vitro* and producing mature and developmentally competent metaphase II oocytes is one of the most challenging topics in reproductive biology. Success in this endeavour would enable the use of the huge ovarian reserve of dormant primordial ovarian follicles, most of which are destined for atresia during the female reproductive life. This reserve of oocytes can be used for fertility preservation and *in vitro* fertilization (IVF) procedures in human- and animal-assisted reproduction. The first successful *in vitro* production of mature oocytes from primordial follicles in newborn ovaries was achieved in a mouse model more than 20 years ago (Eppig and O'Brien, 1996; O’Brien et al., 2003). This demonstrated that oocytes grown *in vitro* may be fertilized and generate embryos that develop normally and give birth to healthy newborns. In the *in vitro* two-step procedure of Eppig and colleagues, different static bioreactors were used to culture whole ovarian tissue strips and isolated preantral follicles until the antral stage. Today, it is generally agreed that primordial follicle activation and development to the multilaminar preantral stage is more effectively performed *in situ*, i.e., inside small strips of intact ovarian tissue (Yang et al., 2020), than by culturing isolated primordial follicles. Application of the same procedure to humans has been, and still is, not successful, possibly for the anatomical and functional differences between the ovaries of small newborns (as was the case in mice) and large adult mammals (as is the case in bovine or humans) that have been used. Recently, McLaughlin et al. (2018) and Xu et al. (2021) have reported the development of human oocytes from primordial follicles to resumption of meiosis with a fully *in vitro* four-step and two-step culture technique, respectively. Such studies provide the proof of concept that it is indeed possible to replicate in humans, the multi-step procedures that are similar to those used in mice. However, McLaughlin et al. (2018) and Xu et al. (2021) have reported that only nine apparently abnormal and three apparently normal metaphase II oocytes could be obtained after 10 and 13 attempts, respectively.

The analysis of published culture experiments (Catapano et al., 2019) suggests that bioreactors currently used do not generate an optimal microenvironment for the *in vitro* culture of viable and functional ovarian tissue. In particular, the bioreactors used for the first ovarian tissue culture step seem more likely to limit the progression and quality of secondary follicles and act as a barrier to the clinical use of the multi-step technique.

Ovarian tissue strips and other ovarian tissue (e.g., cumulus-oocyte complexes, oocytes, or embryos) are generally cultured *in vitro* in static batch bioreactors, such as a conventional culture dish (CD) or modifications thereof (Smitz et al., 2010). Such bioreactors lack a continuous supply of nutrients and oxygen and do not provide continuous removal of waste metabolites. These limitations may generate concentration gradients of oxygen, nutrients, and waste metabolites that make the perifollicular (vs. peri-oocytic or peri-embryo) concentrations of dissolved oxygen and nutrients significantly lower and that of waste metabolites significantly higher than they are in the medium bulk. Moreover, *in vitro*, the transport of dissolved gases and metabolically relevant solutes (e.g., nutrients, waste metabolites, etc.) between ovarian tissue and the medium bulk occurs over far greater distances than they do in natural tissue due to the absence of vasculature. As for oxygen, ovarian tissue is generally cultured under a static layer of medium, often overlaid with an oil layer, and oxygen is supplied from the gaseous atmosphere above the liquids. The liquid layers offer a significant resistance to oxygen transport between the gas/liquid interface and the outer tissue surface (i.e., external to the tissue); the thicker the liquid layers and greater the rate at which the tissue consumes oxygen, the greater becomes the resistance (Catapano et al., 1996). In the culture of strips of ovarian tissue, the high oxygen metabolic consumption of ovarian cells (Fraser et al., 1973) and the poor ovarian tissue permeability (Devireddy, 2005; Fernandez et al., 2013; Rohanifar et al., 2022) make the resistance to oxygen transport internal to the tissue very high. The compound effect of these resistances is that the dissolved oxygen concentration near the follicles may be much lower than it is at the gas/liquid interface that may cause hypoxic or even anoxic conditions in their culture, thus limiting long-term follicle development and viability. In recent times, dynamic bioreactors of macro- to microscales have been proposed for ovarian tissue culture that mimic the continuous supply of nutrients and oxygen (and waste metabolites removal) enabled by the blood circulation in natural tissues (da Rocha and Smith, 2014; Heise et al., 2009; Sadeghzadeh Oskouei et al., 2021; Del Valle et al., 2022; Mastrorocco et al., 2022). Bioreactor designs for ovarian strip culture have been proposed to decrease the liquid resistance to oxygen transport external to the tissue strips in which the medium is mechanically or fluid dynamically mixed, apparently with inconsistent results (Hovatta et al., 1997; Wright et al., 1999; Isachenko et al., 2006; Liebenthron et al., 2013). The analysis of oxygen transport in the context of bioreactor geometry and operation, and the strips' geometry, has demonstrated that the effective decreasing external oxygen transport resistance consistently enhances follicle development and viability (Catapano et al., 2019). This is in agreement with the outcome of ovarian tissue culture experiments performed under decreasing external resistances to oxygen transport in the tissue (Connolly et al., 2019) and the general concept that an optimal bioreactor should prevent prolonged anoxia anywhere in the construct (Malda et al., 2007; Potier et al., 2007). Recently, we provided evidence that a range of dissolved oxygen concentrations inside the tissue exists that may foster follicle development and viability in strips of cortical ovarian tissue (Talevi et al., 2018).

The recent awareness that biochemical and biomechanical stimuli influence morphogenesis through bidirectional coordinated interactions (Matsuzaki, 2021) has drawn attention to the possible role of biomechanical stimuli in the activation and growth of dormant primordial follicles. In fact, the transduction of mechanical stimuli to biological signals (i.e., mechanotransduction) has been shown to influence the metabolism and development of many healthy and cancerous human cells. Tensile or compressive solid stress, fluid dynamic shear stress, pressure, and extracellular matrix (ECM) stiffness have all been reported to cause alterations to cell proliferation, differentiation, and survival in cell-specific fashion (Zhu et al., 2000; Becquart et al., 2016; Kalli and Stylianopoulos, 2018; Novak et al., 2018; Wang et al., 2020; Zonderland and Moroni, 2021). In particular, Benham-Pyle et al. (2015) and Fletcher et al. (2018) have shown that mechanical stretching induces the activation of monolayers of densely packed Madin-Darby canine kidney (MDCK) epithelial cells adherent on compliant silicone substrates and of *Drosophila* follicle cells, respectively, and have elucidated some underlying metabolic pathways. Telfer and McLaughlin (2017) have also reported that stimulating strips of ovarian cortical tissue of large mammals with a short (at least) 10% tensile strain prior to culture promotes follicle growth and enhances their health and viability in subsequent cultures. In all these cases, stretching is believed to cause relaxation of the pressure exerted by the dense cell aggregate on MDCK cells or by the granulosa cells and the stiff ECM of the ovarian cortex on the oocytes that keeps cells and primordial follicles quiescent. These suggestive lines of evidence made us hypothesize that a bioreactor design generating a microenvironment that exploits ovarian tissue response to suitable biochemical and biomechanical signals might promote follicle development and health in *in vitro* cultures of ovarian cortical tissue strips.

In this review, we report on the development and preliminary evaluation of a novel dynamic bioreactor for the culture of strips of ovarian cortical tissue in which the transport of dissolved oxygen between the medium bulk and the strip surfaces is enhanced by continuously perfusing the strips with medium. The strips are also subjected to a steady biomechanical stimulation, in terms of fluid mechanical shear stresses, effected by medium perifusion, and solid compressive strains, effected by squeezing tissue which should relax the pressure exerted *in situ* on primordial follicles by the ovarian granulosa cells and ECM. Digital representations of suitable bioreactor configurations were prepared with computer-aided design (CAD) software, and their design was optimized with 3D models of medium flow and oxygen transport to maximize strip perifusion and to exert biomechanical stimuli possibly eliciting tissue biochemical response. Prototypes of the most promising dynamic bioreactor design (hereinafter referred to as the “final configuration”) were manufactured with materials tested for their cytotoxicity with bovine spermatozoa. Preliminary long-term culture experiments with fresh bovine ovarian cortical tissue strips in such bioreactors yielded significantly enhanced follicle development and viability over static culture, suggesting the goodness of the design approach. Bioreactors made of more cytocompatible materials, such as polypropylene, fostered follicle activation and growth to a greater extent than those made of poorly cytocompatible materials, such as ABS-like resin. Such an outcome shows that the quick sperm-based test predicts well the material cytocompatibility for ovarian tissue, which strongly influences the effectiveness of tissue culture in the novel bioreactor.

## 2 Materials and methods

Some materials and methods used for this study are very similar to those used in other studies published by our group (Talevi et al., 2018; Barbato et al., 2023), and their description may occasionally reproduce verbatim that has been previously reported.

### 2.1 Consumables

Conventional culture dishes that are 50 mm in diameter were purchased from Falcon (Sigma-Aldrich, Milan, Italy). Leibovitz’s L-15 medium, α-MEM GlutaMAX medium, insulin transferrin selenium (ITS) 100×, and LIVE/DEAD Fixable Far Red stain were purchased from Invitrogen (Milan, Italy). Penicillin-streptomycin 100×, amphotericin B 250 μg/mL, bovine serum albumin, L-ascorbic acid, L-glutamine 200 mM, Hoechst 33342, fructose, and α-thioglycerol were purchased from Sigma-Aldrich (Milan, Italy). Mayers’s hematoxylin and paraffin wax were purchased from Carlo Erba (Milan, Italy).

### 2.2 The perifusion bioreactor

#### 2.2.1 Bioreactor design

This study aimed to design a bioreactor in which tissue is cultured in a microenvironment that exploits ovarian tissue response to both biochemical and biomechanical signals. In particular, the novel bioreactor was conceived to enable the effective transport of medium-borne dissolved oxygen to the tissue and the mechanical stimulation of the tissue strips that elicits tissue biochemical response and activates the quiescent primordial follicles by relaxing the pressure exerted on them by granulosa cells and the ECM. Additional requirements were that the bioreactor design had to enable easy loading of tissue strips in fixed space positions and recover them at the end of culture, and use commercial parts and accessories. Meeting these last requirements would facilitate bioreactor use and make it more sustainable.

In the first phase of the work, dynamic bioreactors were considered that had been proposed to enhance oxygen transport to ovarian tissue culture over static bioreactors. In these bioreactors, the medium generally flows around ovarian tissue strips loaded in a conventional culture dish and the resistance to oxygen transport external to the tissue is decreased by operating at a high medium flow rate or by mechanical agitation (e.g., by positioning the dish on an orbital shaker). In a few cases, mixing the flowing medium effectively enhanced oxygen availability thus enhancing follicle viability, health, and development (Catapano et al., 2019), and likely challenged the tissue with fluid dynamic shear stresses. However, both transport enhancement and shear stresses are difficult to evaluate quantitatively, non-reproducible, and beyond the operator’s control. Recently, microfluidic bioreactors have been proposed for ovarian tissue culture although with unsatisfactory effects on *folliculogenesis* (Del Valle et al., 2022). None of the proposed bioreactors permits the application of a direct mechanical challenge on the tissue. In the second phase, bioreactors were considered among those proposed to engineer tissues (Freshney et al., 2007), in particular those conceived to minimize the resistance to solute transport external to, or to exert a mechanical challenge on, tissue constructs. Some bioreactors of the former type successfully enhanced oxygen transport to the constructs and permitted the use of small tissue constructs. However, generally, they did not permit the application of direct mechanical stimuli to constructs (e.g., the rotary wall or the spinner flask bioreactor). Bioreactors of the latter type permit the application of a direct mechanical challenge to the tissue, but they generally use cumbersome tools that can significantly hinder oxygen supply to small-sized constructs. Even those that can also enable oxygen transport to tissues are complicated to build to treat small constructs, making it difficult to reliably apply forces to multiple tissue constructs with one compressive head, and are thereby susceptible to rupture and contamination (Watson and Mikos, 2023).

**FIGURE 1:**
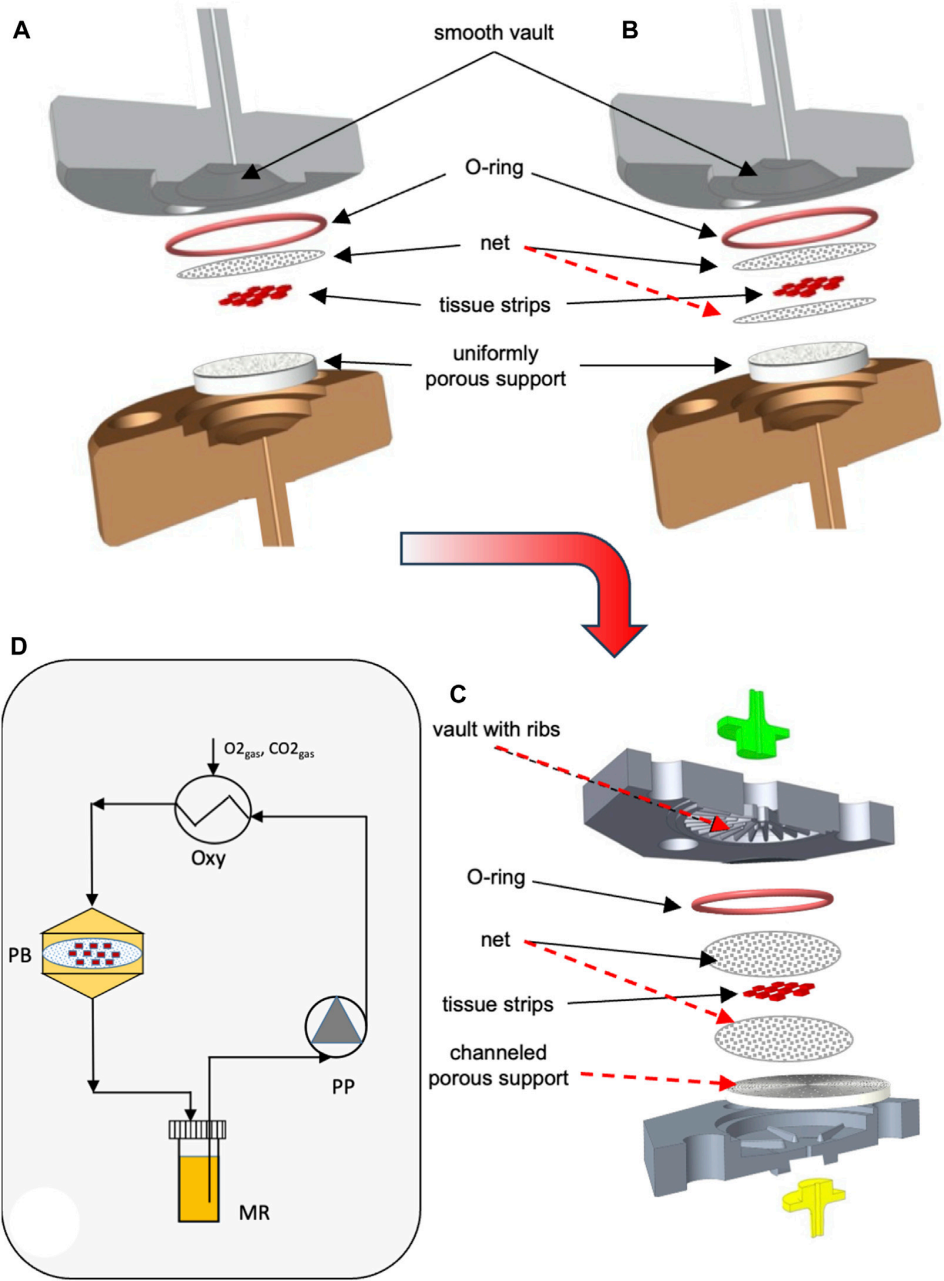
Scheme of bioreactor configurations and experimental apparatus. Scheme of the equipment used for the study: **(A)** dynamic bioreactor in which the tissue strips are overlaid with an open-mesh net and laid on a uniformly porous support; **(B)** dynamic bioreactor in which the tissue strips are enveloped between two open-mesh nets and laid on a uniformly porous support; **(C)** final configuration of the dynamic bioreactor, in which ribs protrude from the vault of the inlet bioreactor half, the tissue strips are enveloped between two open-mesh nets, and channels below the strip bottom are 3D-printed or carved in the top surface of the porous support; **(D)** experimental apparatus used for the culture experiments with dynamic bioreactors: MR, medium reservoir; Oxy, medium oxygenator; PB, perifusion bioreactor; PP, peristaltic pump. The red dashed lines indicate the most relevant features that have been added to the reference configuration (i.e., Figure 1A) as bioreactor development progressed towards the final configuration (i.e., Figure 1C).

For the above reasons, in the third and final phase of the work, a step-by-step approach was followed to develop a novel bioreactor that would use the best approaches of the bioreactors proposed thus far, had no moving parts, and would meet the requirements of this specific application. The development of the novel bioreactor started from a reference dynamic bioreactor design consisting of a culture chamber interposed between two conical input/output halves, in which ovarian tissue strips are laid on a porous support and held in the culture chamber by means of at least one highly permeable net. The two halves may be tightened together by means of screws. Medium is continuously fed to the bioreactor inlet half, enters the bioreactor, and mainly flows downward perpendicular to the larger surfaces of the tissue strips, and around them, filters across the porous support on which the strips are positioned, and then leaves the bioreactor (i.e., a perifusion bioreactor, PB). A gasket (e.g., an O-ring) prevents medium leakage. The most promising bioreactor configuration for enabling both an enhanced oxygen supply to the tissue and a fluid dynamic stimulation eliciting ovarian tissue response was sought by investigating the effect of design variables such as the number and position of the strips, the size of inlet/outlet tubing, the superficial filtrate flux, the structure and permeability of the porous support, and the number and position of the nets, as schematically shown in Figures 1A–C. Eventually, the geometry of the most transport-effective configuration was modified so that the top and bottom surfaces of the tissue strips were subjected to a direct normal solid compressive deformation while the tissue is unconstrained and may freely stretch in the other coordinate directions. Figure 1C shows a sketch of the most promising bioreactor configuration that was developed in this study (hereinafter referred to as the “final bioreactor configuration”). A detailed description of the final bioreactor configuration and its peculiar features may be found in Catapano et al. (2022). Figure 1D schematically shows the setup used for the dynamic culture experiments.

#### 2.2.2 Transport modelling

Mathematical modelling of medium and dissolved oxygen transport was extensively used to optimize bioreactor design. Digital representations of the considered perifusion bioreactor configurations were prepared with the commercial CAD software PTC Creo version 8 (PTC, Boston, MA, United States). The mathematical modelling of occurring transport and metabolic phenomena is a convenient and sustainable approach to the development of an effective bioreactor design (Eibl et al., 2009; De Napoli et al., 2014; Fragomeni et al., 2019). In this study, we focused on medium and dissolved oxygen transport for the metabolic importance of the latter for ovarian tissue. Its properties and effects on the ovarian tissue, as reported in the literature, make it a feasible exemplary metabolically relevant solute to assess the effect of transport enhancement on ovarian tissue.

**FIGURE 2:**
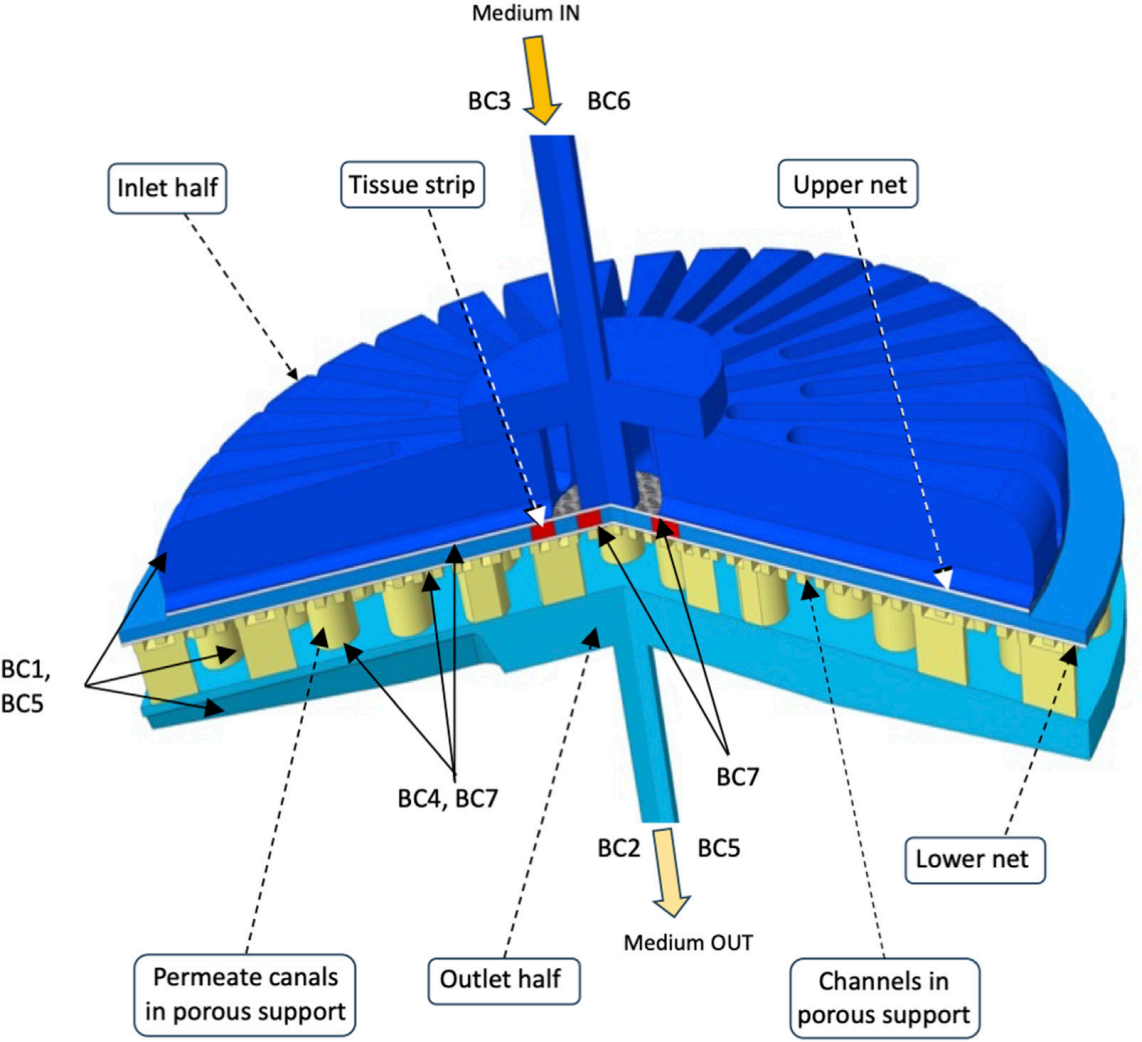
Integration domains in the final perifusion bioreactor. The scheme shows the liquid integration domains of the transport models in the three compartments (i.e., bioreactor inlet and outlet half, nets, and porous support with carved channels) of the final perifusion bioreactor (Figure 1C). The boundaries are also shown, and the conditions that were set on each of them are reported in text as BCs. The integration domains for other considered bioreactor configurations may be obtained by substituting a uniformly porous support to that shown featuring channels and permeate canals (e.g., Figure 1B), and by additionally removing the lower net below the tissue strips (e.g., Figure 1A). Details can be found in Section 2.

Three-dimensional (3D) models of medium flow and dissolved oxygen transport were developed to predict the distribution of medium velocity, shear stresses, and dissolved oxygen concentrations anywhere in any considered bioreactor configuration, in particular at the strip surfaces. Figure 2 schematically shows the empty bioreactor compartments (i.e., bioreactor inlet and outlet half, one or two nets, and porous support where applicable) represented as fluid domains, and the boundaries where the conditions were set that the variables determining bioreactor behaviour have to meet. Medium flow in every empty bioreactor compartment was described according to the Navier–Stokes equations (Bird et al., 2002) for an aqueous incompressible medium. Medium flow through, or in, the nets and the uniformly porous support was described in terms of the Darcy–Brinkman equation for an isotropic porous slab with uniform porosity and Darcy permeability (Brinkman, 1949), or the Navier–Stokes equation for the liquid domains describing in detail the voids in the porous support, as follows:

Balance equations in bioreactor void spaces

(1) $\rho \left(u∙\nabla \right)u=-\nabla p+\mu \nabla^{2}u+\rho g$ , 

Navier–Stokes equation for flow transport,

with ∇∙u=0

continuity.

Balance equations in porous media

(2) $+\nabla p+\frac{\mu }{\kappa_{j}}u-\nabla ∙\left(\mu \nabla u\right)=0$ , Brinkman–Darcy equation

with ∇∙u=0 continuity.

The following boundary conditions (BCs) completed the description of flow transport: BC1, no-slip at the solid surfaces of bioreactor housing and porous support; BC2, atmospheric pressure at the bioreactor outlet; BC3, set medium feed flow rate at bioreactor inlet with fully developed parabolic laminar flow; BC4, continuity of velocity and fluxes at fluid compartment interfaces.

Dissolved oxygen transport in every empty bioreactor compartment and in tissue was described according to the diffusion–convection and diffusion–reaction equations, respectively, as follows (Bird et al., 2002):

(3) $\nabla ∙\left(D_{oxy,m}\nabla C_{oxy}\right)-\nabla ∙\left(uC_{oxy}\right)=0$ , 

convection–diffusion balance equation for oxygen transport in empty compartments

(4) $\nabla ∙\left(D_{oxy,J}\nabla C_{oxy}\right)-\nabla ∙\left(u{\;C}_{oxy}\right)=0$ , 

convection–diffusion balance equation for oxygen transport in porous media, with *j* = *net*, *P*S

(5) $-\nabla ∙\left(D_{oxy,tiss}\nabla C_{oxy}\right)=\frac{V_{\max,oxy}\;C_{oxy}}{K_{m,oxy}+C_{oxy}}$ , 

diffusion–reaction balance equation for oxygen transport in ovarian cortical tissue.

The following boundary conditions (BCs) completed the description of oxygen transport: BC5, oxygen-impermeable solid surfaces; BC6, oxygen concentration in the entering medium in equilibrium with gaseous air; BC7, continuous oxygen concentrations and fluxes at compartment fluid interfaces. The medium entering the PB or at the medium/gas interface in CD was assumed to be in equilibrium with air containing 21% oxygen.

The resulting balance equations were solved numerically with the commercial finite element method software COMSOL Multiphysics ver. 5.3a (Burlington, MA, United States) with a 10^–6^ percent convergence error for each residual component (e.g., velocity components, oxygen concentration in the stream leaving the bioreactor, etc.), for the parameter values reported in Table 1, unless otherwise stated. The computational grid was generated by partitioning the integration domains with an unstructured mesh with tetrahedral elements, the number and density of which were optimized for the specific conditions under which the simulation was performed. Exemplary parameter values were used for the difficult reliable estimation of many of them. This is particularly true for the porosity and Darcy permeability of porous support and nets. The complex (at times ill-defined) and broadly diverse characteristics of the structures that were considered in bioreactor development made it impractical to estimate their value with phenomenological generic Carman–Kozeny type equations (Chapuis and Aubertin, 2003) or modifications thereof developed for specific porous structures (e.g., as in Guazzelli et al., 2023). Hence, these parameter values were inferred from those of tissue engineering scaffolds accounting for possible structural differences. The digital CAD description of the bioreactor components was optimized for conversion to the STP format with the commercial CAD software PTC Creo version 8 (PTC, Boston, MA, United States) and imported in COMSOL as the integration domain.

**TABLE 1:** Parameter values used for model predictions, unless otherwise stated.

Parameter	Value	Units	Description	Reference
*C*_*oxy,in*_	0.216	mol/m^3^	Inlet oxygen concentration	Han and Bartels, 1996
*D*_*oxy,m*_	3.5∙10^–9^	m^2^/s	Oxygen diffusivity in medium	Place et al., 2017; Peniche Silva et al., 2020
*D*_*oxy, tiss*_	2.8∙10^–9^	m^2^/s	Oxygen diffusivity in tissue	Catapano et al. (2019)
*J*_*F*_	4.8∙10^–5^	m/s	Superficial filtrate flux	This study
*K*_*M,oxy*_	5∙10^–3^	mol/m^3^	Michaelis constant for oxygen consumption	This study
*k*_*net*_	10^–6^	m^2^	Darcy permeability of nets	This study
*k*_*PS*_	10^–8^	m^2^	Darcy permeability of uniformly porous support	This study
*R*_*in*_	1.5∙10^–3^	m	Radius of inlet/outlet tubing	This study
*V*_*max, oxy*_	2∙10^–2^	mol/(s∙m_*tiss*_^3^)	Maximal oxygen consumption rate	Kim et al. (2004)
*δ*_*tiss*_	6∙10^–4^	m	Strip thickness	This study
*ε*_*net*_	0.9	—	Porosity of the nets	Karageorgiou and Kaplan, 2005; Ochoa et al., 2009
*ε*_*ps*_	0.9	—	Porosity of porous support	Karageorgiou and Kaplan, 2005; Ochoa et al., 2009
*μ*_*m*_	8.9∙10^–4^	kg/(m∙s)	Medium viscosity	Poon (2022)
*ρ*_*m*_	1,000	kg/m^3^	Medium density	Poon (2022)

To promote oxygen transport from the medium bulk to the follicles inside the ovarian tissue, the dissolved oxygen concentration decreases to overcome the resistance to transport external and internal to the tissue. The former is associated with medium flow at the medium/tissue interface, while the latter is associated with oxygen metabolic consumption and diffusive transport inside the tissue. Information about the extent of oxygen concentration decay internal to a tissue strip and external to the tissue was gathered from the values of the dimensionless Thiele modulus, *ϕ*_*oxy*_, and Biot number for oxygen transport, *Bi*_*m,oxy*_ = *k*_*c,oxy*_ *δ*_*tiss*_ */D*_*oxy,tiss*_. *ϕ*_*oxy*_ compares the rate of metabolic oxygen consumption to oxygen diffusion inside the tissue. *Bi*_*m,oxy*_ compares the rate of the medium bulk to tissue oxygen transport external to the tissue with oxygen diffusive transport inside the tissue. As such, *Bi*_*m,oxy*_ provides an estimate of the concentration decay ratio inside the tissue and external to it. *k*_*c,oxy*_ is the reciprocal resistance to medium bulk to tissue oxygen transport and is estimated according to the “film model” as *k*_*c,oxy*_ = *D*_*tiss*_/*δ*_*sl*_. *δ*_*sl*_ is the thickness of the stagnant medium layer adherent on the tissue surface over which oxygen is assumed to diffuse and over which oxygen concentration decreases from 99% of the bulk value to that at the medium/tissue interface (Bird et al., 2002).

#### 2.2.3 Bioreactor manufacture

The final configuration bioreactors were designed and manufactured such that they permitted the use of accessories (e.g., porous supports and O-ring) commercially available for 25-mm and 47-mm-diameter filter supports. The bioreactor design was scaled with respect to the superficial filtration flux. The bioreactors used for the preliminary culture experiments featured a 6.9-mL inner culture chamber, a 47 mm inner diameter, ribs protruding from the inlet and the outlet vaults, concentric and communicating channels carved on the top surface of the porous support, and 1.5-mm-large inlet and outlet tubing. The ovarian tissue strips were held in the culture chamber sandwiched between two multifilament woven medical-grade polyester nets with 330 × 290 μm large meshes. A medical-grade silicon O-ring was used as the gasket. The bioreactor prototypes for ovarian cortical tissue culture were manufactured with selected materials, as described below, by specialized third parties on the authors’ specifications or were prepared in the authors’ workshop by suitably modifying devices developed for other applications to match the specific design requirements.

### 2.3 Characterization of material cytocompatibility

#### 2.3.1 Sample preparation

Prior to their use for bioreactor preparation, materials were screened for their cytotoxicity for ovarian tissue. Both certified biomaterials [i.e., food-grade polypropylene (PP), and the medical-grade untreated polyamide (uPA), polycarbonate (PC), and dental photoreactive resin (DR)] and technical materials [i.e., ABS-like resin (ABS-L-R), surface-treated polyamide (stPA), basic and professional technical resins (BTR and PTR, respectively), and stainless steel (SS)] were selected as potential candidates. Material samples were either kindly provided by the manufacturer or prepared in the authors' workshop. The latter were 3D printed by stereolithography (SLA) with the Zortrax Inkspire 3D printer (Zortrax, Olsztyn, Poland), cured for at least 8 h in direct sunlight, and tested without further post-treatment. Prior to testing their cytocompatibility, the material samples were dipped twice in a solution containing 1% (v/v) of the commercial detergent LM1 (Kartell S.p.A., Labware Division, Milan, Italy) in DI water for 30 min in an ultrasonic bath (Mazzali Srl, Reggio, Italy) at *T*_room_. Then, the samples were carefully rinsed with DI water for 30 min and dried in an air stream under a laminar flow hood for 30 min. The samples were then sterilized by incubation in 2% (v/v) glutaraldehyde for 20 min at T_room_, followed by thorough rinsing with sterile DI water.

#### 2.3.2 Cytocompatibility assessment

Material cytocompatibility with ovarian tissue was characterized with respect to leachable toxic species. Leachables were extracted by incubating the sterile material samples in M199 culture medium (Sigma-Aldrich, Milan, Italy) at a sample surface area to medium volume ratio of 3 cm^2^ mL^−1^ for 72 h at 37°C. The cytotoxicity of leachables was assessed with respect to the motility and viability of bull spermatozoa. In brief, bovine spermatozoa straws (Intermizoo, Padova, Italy) were thawed at 37°C, washed with M199 medium supplemented with 0.6% BSA, 1% penicillin–streptomycin, 1 mg/mL amphotericin B, centrifuged 10 min at 170*g*, and the cryoprotectant-containing supernatant was discarded. Then, 2∙10^6^ bull spermatozoa were incubated with 1 mL of the leachable-containing medium for 1–5 h at 37°C. Every hour, 10 μL of the spermatozoa suspension was harvested, and spermatozoa motility was characterized. The culture medium that had not contacted the material samples was incubated at 37°C for 72 h and served as the control. The number of total and progressive motile spermatozoa was assessed by examining every spermatozoa-containing sample in a Makler chamber on a preheated stage at 37°C under a Nikon Eclipse TE2000 inverted microscope (Nikon, Amstelveen, the Netherlands) connected to a Basler Vision Technology A312 fc camera with a positive phase contrast ×10 objective. The Sperm Class Analyzer (SCA) (Microptic S.L., Barcelona, Spain) software was used to characterize spermatozoa kinetics, in terms of curvilinear velocity (VCL), straight-line velocity (VSL), and average path velocity (VAP); settings: frame rate = 25 frames/s; frames/object = 10; 50% minimal linearity; 80% straightness for progressive fast spermatozoa. Sperm velocity was analysed by grouping the spermatozoa into four categories featuring motility within ranges based on the bull-specific VAP cut-off, as outlined in the SCA system v.3.4.0, as follows: rapid cells (percent spermatozoa featuring VAP >50 μm/s, labelled *rapid)*; medium-speed cells (percent spermatozoa featuring VAP between 25 and 50 μm/s, labelled *medium*); slow-moving cells (percent spermatozoa featuring VAP between 10 and 25 μm/s, labelled *slow*), and static cells (percent spermatozoa not moving at all, labelled *static*). Spermatozoa motility at the initial time point served as the control.

### 2.4 Characterization of the solid compressive strain

The shape and depth of the solid compressive strains exerted on tissue by the concerted action of ribs, nets, and porous support geometry was characterized with respect to model strips made of the orthodontic wax for braces Cliadent (Budetta Farma s.r.l., Montecorvino Pugliano (SA), Italy. A polylactic acid template with cylindrical or rectangular cavities was 3D-printed by fused deposition modelling. The model strips were prepared by filling the cavities in the template with wax with a heated scalpel and by carefully peeling them off once they had cooled down and solidified. The procedure yielded cylindrical (1.5 mm in diameter) or slab (5 × 7 mm large) wax samples, 0.6–0.65 mm thick. Their geometry was checked to be regular prior to their use. Selected model strips were loaded in the bioreactor according to the same procedure as the tissue strips, and the bioreactor was securely tightened. After 5 min, the bioreactor was opened, the model strips were recovered, and the strains at their surfaces were analysed (*n* = 10) with an Rtec Instruments Universal 3D Profilometer UP Series (Rtec, B San Jose, CA, United States) in the confocal mode using either a BF-5X or BF-10× confocal lens. Image brightness was adjusted to a 30%–40% hue level. Image analysis of high-quality surface scans was performed to characterize the depth of randomly chosen strains along two equally spaced lines and the contact surface area between a tissue strip and net. To this purpose, software such as the proprietary Rtec Lambda 18R1, the open-source Gwyddion SPM (scanning probe microscopy) (http://gwyddion.net), and the Creo version 8 (PTC, Boston, United States) were used. The maximal total solid compressive strain was estimated as the ratio of the sum of the maximal residual plastic strains on the top and bottom surfaces of each sample to the sample's initial thickness.

### 2.5 Ovarian tissue strip preparation

Bovine ovaries were obtained from the slaughterhouse Straccione (San Marcellino, Caserta, Italy; CEE accreditation number 1403/M) when the animals were eviscerated and were transported within 2 h to the laboratory in Leibovitz's L-15 medium, supplemented with 1% penicillin–streptomycin (Pen-Strep) and 1 μg/mL amphotericin-B, kept at 4°C.

Cortical tissue slices were reliably dissected from the bovine ovaries with a custom-made tissue slicer and were soaked in handling medium (i.e., Leibovitz's L-15 medium supplemented with 2 mM glutamine, 3 mg/mL BSA, 1% Pen-Strep, and 1 μg/mL amphotericin B) at T_room_, discarding zones containing antral follicles. Then, 1 mm × 1 mm large, 0.6 mm, i.e., 588 ± 56-μm-thick (Barbato et al., 2023) cortical tissue strips were reproducibly prepared with a tissue chopper (Mcilwain, Mickle Laboratory Engineering Company, Ltd., Surrey, UK). Then, the strips were rinsed twice in fresh handling medium and were used for the culture experiments.

### 2.6 Culture experiments

Ten tissue strips were randomly selected and loaded for culture into either the novel dynamic perifusion bioreactor (PB) or a static conventional culture dish (CD). The tissue strips were positioned at a nominal 1 mm distance from one another at the centre of each bioreactor on three rows in a 3-4-3 staggered pattern. Strips of fresh tissue from each ovary were processed for histology and viability assessment as controls. Culture in CDs was the standard control. α-MEM supplemented with 3 mM glutamine, 0.1% BSA, 1% Pen-Strep, 1% ITS (10 μg/mL insulin, 5.5 μg/mL transferrin, and 6.7 ng/mL selenium), 1 μg/mL amphotericin-B, and 50 μg/mL ascorbic acid was used throughout. Hereinafter, such a culture medium is referred to as medium.

#### 2.6.1 Dynamic culture in the perifusion bioreactor

The setup used for the preliminary dynamic culture experiments is schematically shown in Figure 1D. In brief, a prototype of the perifusion bioreactor was inserted in a test loop in which medium is recirculated with a peristaltic pump, at controlled feed flow rate, from a vented reservoir to a silicon tubing used for exchanging gases between the atmosphere in the incubator and the medium, to the bioreactor, and eventually back to the reservoir. Tubing was purchased from Cole-Parmer (Milano, Italy) and the peristaltic pump from Ismatec (Enco, Venice, Italy). Prior to loading the tissue strips in the bioreactor, the bioreactor was assembled and connected to the experimental setup, and the whole setup was sterilized by circulating 2% (v/v) glutaraldehyde in sterile water for 20 min at *J*_*F*_ = 4.8 × 10^−5^ m/s superficial filtrate flux. Sterile water was recirculated for 25 min to remove any residual glutaraldehyde, the set-up was rinsed with PBS supplemented with 25 μg/mL amphotericin B and 1% Pen-Strep for 20 min, and then rinsed again with sterile water for other 10 min. Then, it was emptied and the bioreactor was disassembled under the laminar flow hood. Ten strips of ovarian cortical tissue from each ovary were randomly selected among those available and were positioned on the porous support around the bioreactor axis at a nominal 1 mm distance from one another, in a 3-4-3 staggered pattern (e.g., as shown in Figure 1C), held in fixed positions between two polyester nets. The two bioreactor halves were tightened together with screws, and the bioreactor was connected again to the recirculation loop. Prior to culture, the setup was primed with pre-heated medium, and the bioreactor was carefully deaerated with an upside-down inversion and gentle shaking. The whole set-up was incubated at 37°C in a 5% carbon dioxide and 95% humidity air atmosphere for culture. Culture experiments were performed by exerting on the strips a maximal −15% steady solid compressive strain and by recirculating medium at *J*_*F*_ = 4.8 × 10^−5^ m/s. Tissue was cultured for 7 days. Half volume of spent medium in the reservoir was replaced with fresh medium every 48 h.

#### 2.6.2 Static culture in conventional dish

Ten strips of ovarian cortical tissue from any given ovary were randomly selected among those available and were loaded and cultured in 5 mL of medium in a conventional culture dish (CD) under the same conditions as the perifusion bioreactor. Half the volume of spent medium was replaced with fresh medium every 48 h.

### 2.7 Analytical methods

At the end of each culture experiment, five strips from the PB and five from the CD were treated for histology. The remaining strips were prepared for viability assessment. The effectiveness of tissue culture in the various bioreactors was assessed in terms of follicle grading, staging, and viability at the end of culture.

#### 2.7.1 Histological analysis

For the histological analysis, the cortical strips were fixed in Bouin’s solution, dehydrated in solutions with serially increasing ethanol concentrations, and then embedded in paraffin and 5-μm serial sections were stained with hematoxylin and eosin. Follicle grading and staging were performed by two blinded expert observers. Only follicles in which the germinal vesicle was clearly visible were classified. Follicle quality was graded as previously described (Talevi et al., 2013). Briefly, grade I follicles are spherical with uniformly distributed granulosa cells (GCs) and an oocyte presenting a homogenous cytoplasm and slightly granular nucleus with clearly visible spherical condensed chromatin; grade II follicles have non-uniformly distributed GCs around a spherical oocyte; and grade III follicles have pyknotic GCs and distorted and/or vacuolized oocyte. Exemplary histological images of follicles of various quality are shown in Supplementary Figure S1. Follicle staging was scored according to Gougeon’s criteria (Gougeon, 1996) as follows: primordial follicles have a single layer of flat GCs, primary follicles have a complete single layer of cuboidal GCs, and secondary follicles have two or more complete layers of cuboidal GCs. Exemplary histological images of follicles at each stage of development are shown in Supplementary Figure S2.

#### 2.7.2 Viability assessment

Tissue viability was assessed by confocal microscopy analysis. The cortical strips were incubated, under shaking, for 3 h at 4°C in Dulbecco’s PBS supplemented with 1 μg/mL LIVE/DEAD Fixable Far red stain and 10 μg/mL Hoechst 33342, were fixed in 4% paraformaldehyde in PBS for 2 h at T_room_, washed in fresh PBS, and incubated at 4°C overnight in PBS supplemented with 10 μg/mL Hoechst 33342 (Talevi et al., 2016). The LIVE/DEAD probe is resistant to fixation, reacts with free amines both in the cell interior and surface, and it is excluded by cells with intact membranes. The strips were then optically cleared using the SeeDB clearing protocol (Ke et al., 2013). In brief, the strips were serially incubated in 5 mL of solutions containing 20%, 40%, and 60% (w/v) fructose, each for 6 h, followed by incubation in 80%, 100%, and 115% fructose (w/v), each for 12 h, all under gentle agitation at T_room_. The fructose solutions were supplemented with 0.5% α-thioglycerol. To avoid compression, the strips were mounted in 115% fructose solution on a glass slide with three spacer coverslips (0.17 mm thick) placed on each side and covered with a coverslip. The strips were analysed under a Leica TCS SP5 confocal scanning laser microscope (Leica Microsystems, Wetzlar, Germany) with a 405-nm diode laser for visualizing the nuclear label (Hoechst 33342) and a 633-nm helium–neon laser for the LIVE/DEAD probe. Each strip was traversed using the z-position control, and fields were imaged to a depth of 300 μm from the tissue surface with a ×63 glycerol immersion objective.

### 2.8 Statistical analysis

Experiments were generally performed in triplicates using different biological materials. Ovarian tissue strips from different zones of the same ovary or from a different ovary, or spermatozoa from different zones of the same straw or from different straws, were used to this purpose. Data are generally reported as mean ± SEM. Data for follicle quality, staging, and viability are reported as cumulative percentages. GraphPad Prism 8.0.2 (263) (GraphPad Software, Boston, MA, United States) was used for statistical analysis of sperm morphokinetics and follicle grading and scoring. Data analysis according to the D’Agostino–Person test showed that, in general, data were not normally distributed. The effect of leachables from various materials on sperm motility parameters and the effectiveness of culture in bioreactors made of various materials were assessed by comparing pairs of materials and bioreactors. To this purpose, the statistical analysis of different outcomes was performed according to Fisher’s exact test for pairwise comparisons.

## 3 Results

The development of the reference bioreactor design into an effective and practical configuration occurred in steps, as schematically shown in Figures 1A–C.

### 3.1 Transport enhancement

**FIGURE 3:**
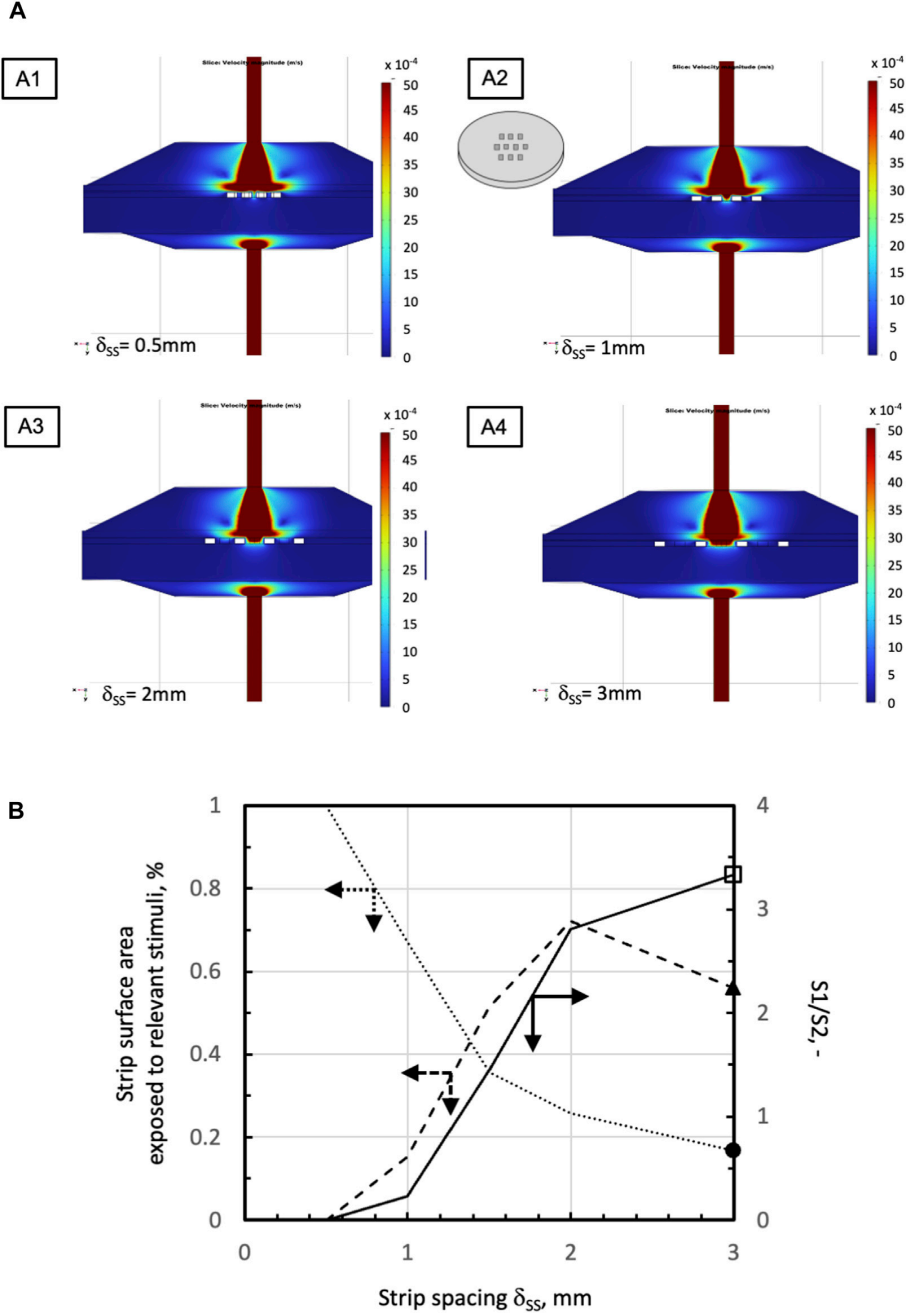
Effect of strip spacing on medium velocity and shear stress distribution in a perifusion bioreactor with one net overlaid on the strips (Figure 1A). **(A)** Model-predicted medium velocity distribution at the middle plane of the perifusion bioreactors in the configurations shown in Figure 1A at *varying strip spacing*: A1, *δ*_*ss*_ = 0.5 mm; A2, *δ*_*ss*_ = 1 mm; A3, *δ*_*ss*_ = 2 mm; A4, *δ*_*ss*_ = 3 mm. **(B)** Model-predicted effect of varying strip spacing on the percent of available total strip top surface area exposed to relevant *stimuli*:
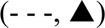
 percent surface area *S1* exposed to shear stresses between 10^–3^ Pa and 10^–2^ Pa;
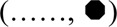
 percent surface area *S2* exposed to medium velocity >1.8∙10^–3^ m/s;
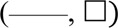
 *S1* to *S2* ratio, *S1/S2*. Simulations performed for the following parameter values: 10 strips in staggered 3-4-3 pattern, *J*_*F*_ = 4.8.10^–5^ m/s, *d*_*tubing*_ = 1.5 mm, *k*_*PS*_ = 10^–8^ m^2^, and *k*_*net*_ = 10^–6^ m^2^. Other parameter values are as in Table 1.

The mathematical models of medium and dissolved oxygen transport were used to seek bioreactor configurations that would enhance oxygen supply as compared to a static conventional dish. In all configurations, an open-mesh net was overlaid on the strips to hold them remain fixed in space, at a preset regular distance from one another. When the strips were laid in direct contact with a uniformly porous support (e.g., a porous membrane) (Figure 1A), the flow model predicted that strips would be better perifused if the medium entered the bioreactor through a large tubing at high flow rates. The bioreactor was equipped with poorly permeable uniformly porous supports, and the strips were laid as close as possible to one another in the middle of the culture chamber. Figures 3A, B show that increasing the space among neighbouring strips decreases the strip surface area effectively bathed by the medium. Figure 3B shows that the strip surface area exposed to shear stresses between 10^–3^ Pa, the threshold above which cells appear to respond biochemically, and 10^–2^ Pa, the threshold above which the tissue may be damaged, attains a maximum at a strip spacing of δ_SS_ = 2 mm. A narrow inlet/outlet tubing and a highly permeable uniformly porous support were preferred for practical reasons. In fact, a narrow inlet/outlet tubing facilitates connection to the recirculation loop and minimizes the loop volume. A highly permeable porous support minimizes the support fouling in long-term culture experiments. Strips were spaced 1 mm from one another (i.e., δ_SS_ = 1 mm) because such spacing would ensure good tissue perifusion and the fluid dynamic stimulation of a significant fraction of the top strip surface (i.e., yielding a ratio close to 1 of the strip surface area subjected to biochemically responsive shear stresses to that well perfused with medium). The model-predicted 3D medium velocity profiles showed that keeping the strip in direct contact with a uniformly porous support results in suboptimal perifusion of the strip bottom. Then, a highly permeable open-mesh net was interposed between the strips and the uniformly porous support (Figure 1B) aiming to facilitate medium flow around and underneath the strips. The flow transport model predicted only minimal enhancements to the strip's bottom perifusion, even at high medium flow rates. However, sandwiching the strips between two nets facilitated strip loading into the culture chamber and recovery at the end of culture, which has been retained in the following developments.

**FIGURE 4:**
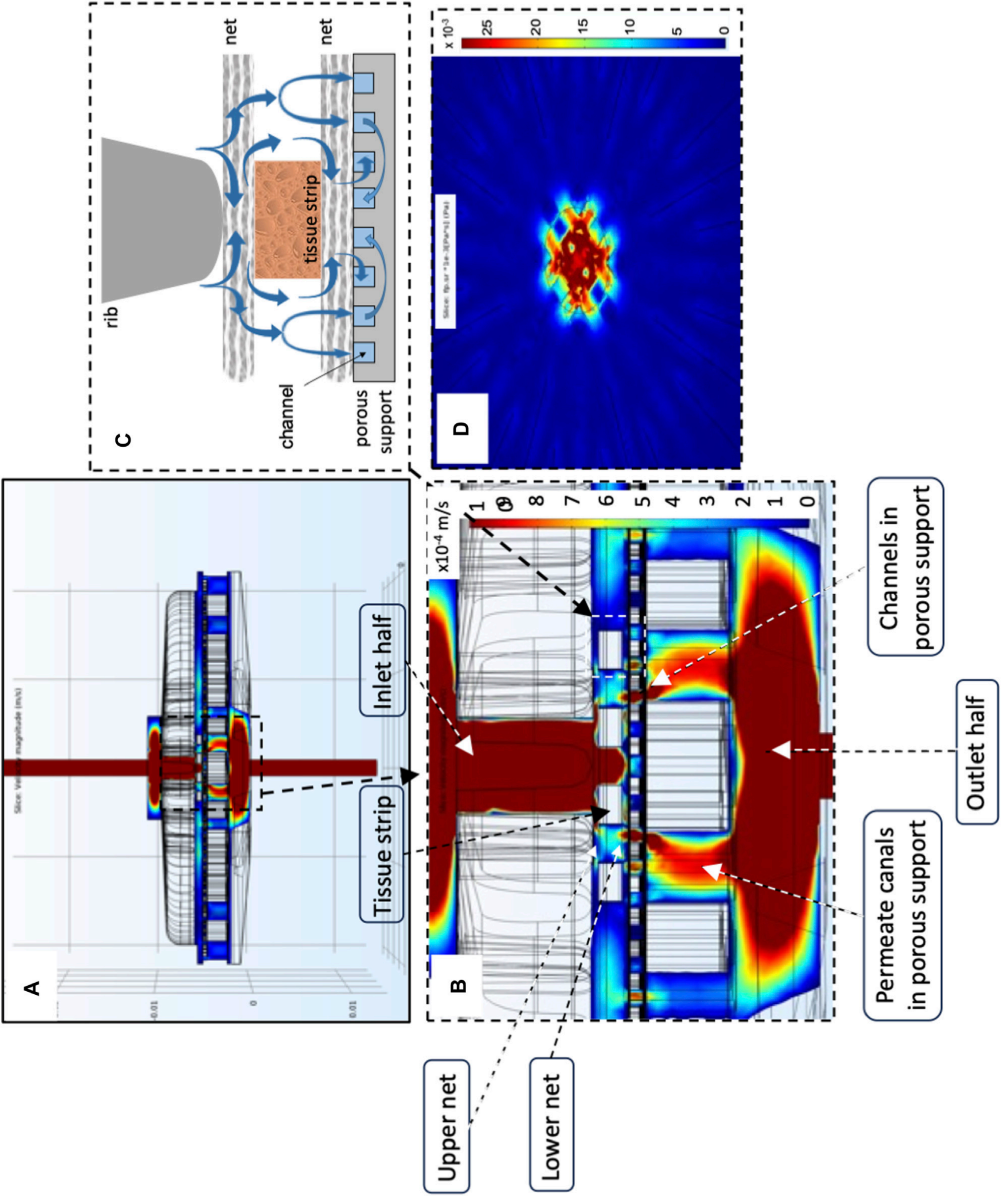
Model-predicted medium velocity and shear stress distribution in the final perifusion bioreactor. Model-predicted medium velocity and shear stress 3D distribution in the culture chamber of the final dynamic bioreactor shown in Figure 1C: **(A)** velocity profiles at the bioreactor middle plane; **(B)** magnification of velocity profile around the tissue strips; **(C)** expected effect of bioreactor components on strip perifusion and mechanical stimulation; **(D)** model-predicted shear stresses on the plane passing through the tissue strip top surfaces and parallel to it. Predictions obtained for *J*_*F*_ = 4.8∙10^–5^ m/s, *d*_*tubing*_ = 1.5 mm, strip pattern and number = 10 staggered strips, *δ*_*ss*_ = 1 mm, and *k*_*net*_ = 10^–6^ m^2^. Other parameter values are as in Table 1.

In the most transport-effective configuration (herein termed final configuration), shown in Figure 1C, the bioreactor design was changed by adding two features to the second net below the strips that were expected to enhance the effectiveness of oxygen transport to the tissue: concentric communicating channels were carved (or 3D-printed) on the top surface of the porous support, on which the strips lay sandwiched between two highly permeable nets, and ribs were added that protrude from the vault towards the porous support in the inlet bioreactor half. The concentric communicating channels were introduced to enhance the strip's bottom surface area that effectively exchanges oxygen with the flowing medium and forces the medium to flow along and underneath the strip surfaces, to bath them, before the medium filters across the porous support and leaves the bioreactor culture chamber (as shown more in detail in Figures 2, 4A–C). The ribs were introduced to guide medium flow towards the strips and prevent the formation of stagnation zones in the culture chamber.

The transport models predicted that the average oxygen concentration at the strip bottom surface would be higher in the final bioreactor configuration (as in Figure 1C) than in all the previous configurations, being on an average 21% higher than in a bioreactor such as that shown in Figure 1A and about six times higher than the hypoxic threshold. The top and bottom strip surfaces are also exposed to closer average oxygen concentrations. In a bioreactor in the final configuration operated at *J*_*F*_ = 4.8^.^10^–5^ m/s, the oxygen concentration at the strip top surface would only differ about 3% from that at the bottom. In comparison, it would differ more than 40% in the bioreactor configuration in which the strips are laid in direct contact with a uniformly porous support. Correspondingly, oxygen availability along a line passing through the middle of a strip positioned near the bioreactor axis (i.e., the average oxygen concentration inside the tissue at that position) is predicted to be 28% higher in the final configuration, and well above the hypoxic threshold.

**FIGURE 5:**
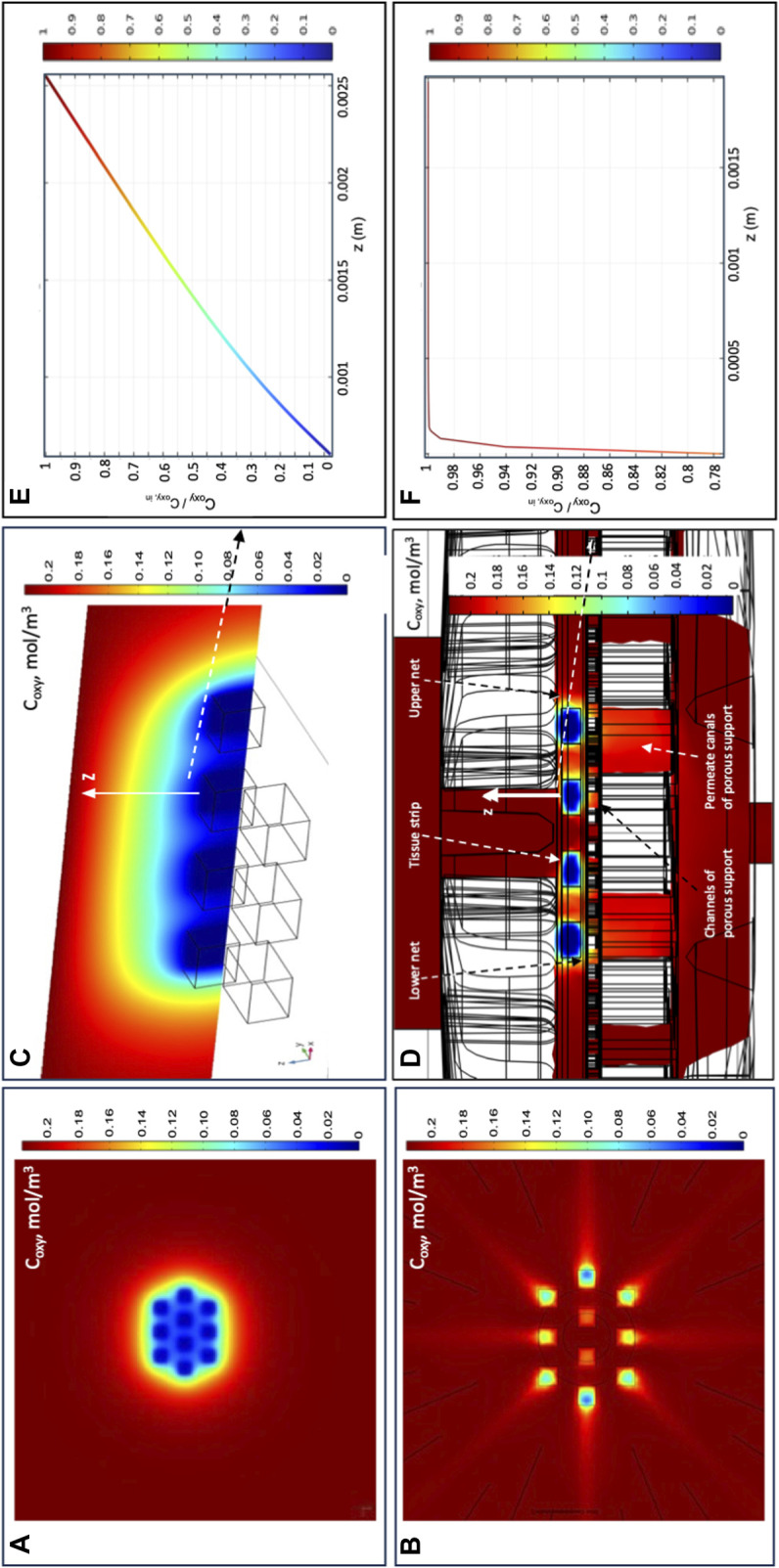
Model-predicted dissolved oxygen concentrations in the final perifusion bioreactor and in static dish culture. Model-predicted 3D profiles of the dissolved oxygen concentration (C_oxy_, mol/m^3^) for 10 tissue strips cultured in: **(A,C,E)** conventional dish CD or in the novel perifusion bioreactor PB **(B,D,F)**—**(A,B)** C_oxy_ profile over the top strip surfaces in **(A)** static dish culture, CD; and **(B)** perifusion bioreactor, PB; **(C,D)** C_oxy_ profile over the symmetry plane passing for the central four tissue strips in **(C)** static dish culture, CD; and **(D)** perifusion bioreactor, PB; **(E,F)** model-predicted C_oxy_ profile normalized with respect to the inlet oxygen concentration (i.e., C_oxy_/C_oxy,in_) along the direction orthogonal to the top strip surface and away from it, positioned at the centre of the strip (white arrow), for strips cultured in **(E)** static dish culture, CD; and **(F)** perifusion bioreactor, PB. Predictions obtained for static dish culture, CD—C_oxy,b_ = C_oxy,in_; perifusion bioreactor, PB—*J*_*F*_ = 4.8∙10^–5^ m/s, *d*_*tubing*_ = 1.5 mm, 10 staggered strips, *δ*_*SS*_ = 1 mm, and *k*_*net*_ = 10^–6^ m^2^. Other conditions are reported in Section 2.

Figures 5A–F show that oxygen transport in the novel perifusion bioreactor is predicted to be far more efficient than in a static culture dish, a bit less so for the strips positioned farther from the bioreactor axis. The model-predicted dissolved oxygen concentration profiles suggest that the enhanced tissue strip perifusion yields also a significant reduction of the resistance to oxygen transport from the medium bulk to the tissue strips. Figures 5E, F show that the model-predicted medium layer above a central strip over which the oxygen concentration decreases is about seven times thicker in a static culture dish than in the novel perifusion bioreactor. As a result, in the novel perifusion bioreactor, the dissolved oxygen concentration around the strips, and at their top surface, is consistently well above the 1%–2% hypoxic threshold whereas that in the static culture dish is predicted to be well below the hypoxic threshold and to establish a close-to-anoxic culture environment (Figures 5A–D). The flowing medium around the strips also permitted to stimulate the tissue with fluid mechanical shear stresses in the 10^−3^–10^–2^ Pa range, a mechanical stimulus missing in the static culture dish.

**FIGURE 6:**
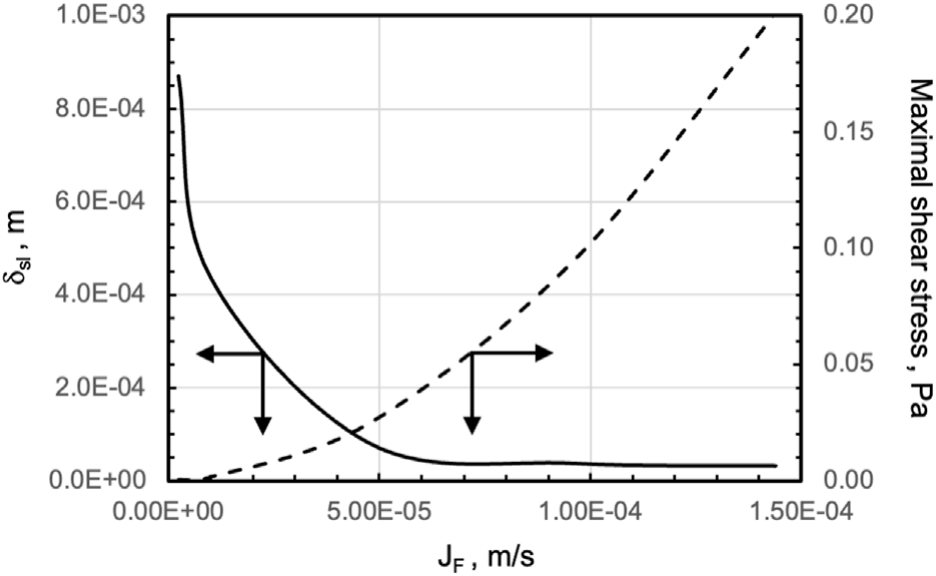
Model-predicted effect of increasing filtrate fluxes on oxygen supply to, and fluid dynamic stimulation of, tissue strips in the final perifusion bioreactor. Effect of increasing filtrate flux, *J*_*F*_, on (solid line, ^____^) the thickness of the medium stagnant layer adherent on the top strip surface at the centre of the strip, *δ*_*sl*_, and (dashed line, - - -) the maximal shear stress, at the upper surface of an exemplary strip. Reference is made to a strip located close to the centre of the four-strip row of a 10 (3-4-3)-staggered strip pattern in the final perifusion bioreactor PB. Other conditions are as in Figure 4.

The transport models were used to predict the bioreactor behaviour at superficial filtrate fluxes varying over two orders of magnitude to understand the extent to which perifusion affects tissue biochemical and fluid-mechanical stimulation. Figure 6 shows that increasing the superficial filtrate flux increasingly reduces the thickness of the stagnant medium layer adherent on a strip and significantly increases oxygen transport to tissue and oxygen concentration at the strip surface. Correspondingly, the strips are subjected to maximal shear stresses that increase from 2.32∙10^–4^ Pa to more than 10^–1^ Pa as the filtrate flux *J*_*F*_ increases from *J*_*F*_ = 2.41∙10^–6^ m/s to *J*_*F*_ = 1.44^∙^10^–4^ m/s, respectively.

### 3.2 Direct mechanical stimulation

**FIGURE 7:**
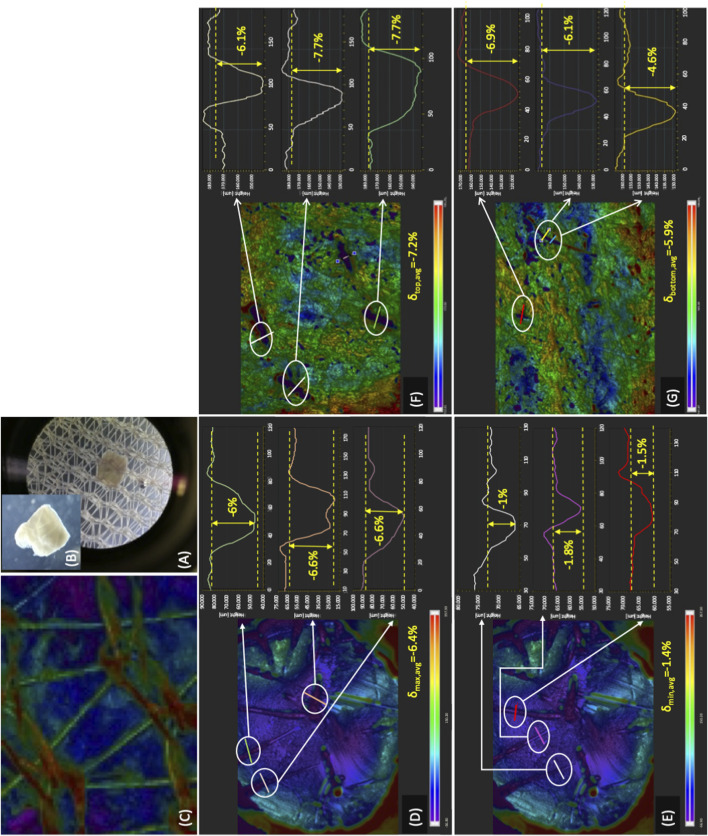
Compressive strains exerted on tissue in the final perifusion bioreactor. Characterization of the compressive strains exerted on tissue in the final dynamic bioreactor (Figure 1C): **(A)** optical microscopic image of a tissue strip on one of the open-mesh nets; **(B)** magnified optical microscope image of a tissue strip showing the compressive strains; **(C)** image of the open-mesh net acquired by 3D profilometry showing filaments and knots present in the net; **(D,E)** characterization of the compressive strains exerted on a wax cylindrical sample by net knots **(D)** and filaments **(E)**; **(F,G)** characterization of the compressive strains exerted by the net knots on the top **(F)** and bottom **(G)** surface of wax mock strips.

Eventually, the height of the ribs in the inlet bioreactor half was dimensioned so that they could exert biochemically relevant direct solid compressive strains on strips of the thickness used for the study (i.e., 0.6 mm). This way, the ribs would not only guide the medium towards the strips but would also enable the direct mechanical stimulation of the strips in concerted action with the two nets enveloping the strips (Figures 1A, Figures 4C). The 3D profilometry effectively enabled the characterization of the strain depth, once artefacts were overcome that had been introduced by the preparation of the wax model strips and by light transmission through the samples. Figure 7A shows that the nets feature an open-mesh woven structure made of filaments and knots, the latter formed and connected to one another by filaments. The solid compressive forces orthogonal to the strip's top and bottom surfaces exerted by the nets effectively caused significant, albeit spatially non-uniform, strains on the tissue (Figures 7A, B). A maximal 32.5% of the available strip surface was in contact with the nets and produced strains the shape and depth of which mirrored the net geometry (Figures 7C–E). Strains were deep with an elongated ellipsoidal shape where the knots contacted the strip and were shallow, thinner, and straight where the filaments contacted the strip (Figures 7D, E). Correspondingly, the strain depth on the top and bottom strip surfaces, respectively, varied from −6.9% ± 1.3% to −8 ± 1%, when the strains were caused by the knots, and from −1.8% ± 0.3% to −2.3% ± 0.4%, when the strains were caused by the filaments. The maximal total solid compressive strain (i.e., the sum of maximal strains on both strip surfaces) averaged approximately −15% of the sample thickness pre-compression.

### 3.3 Cytocompatibility of material candidates

**FIGURE 8:**
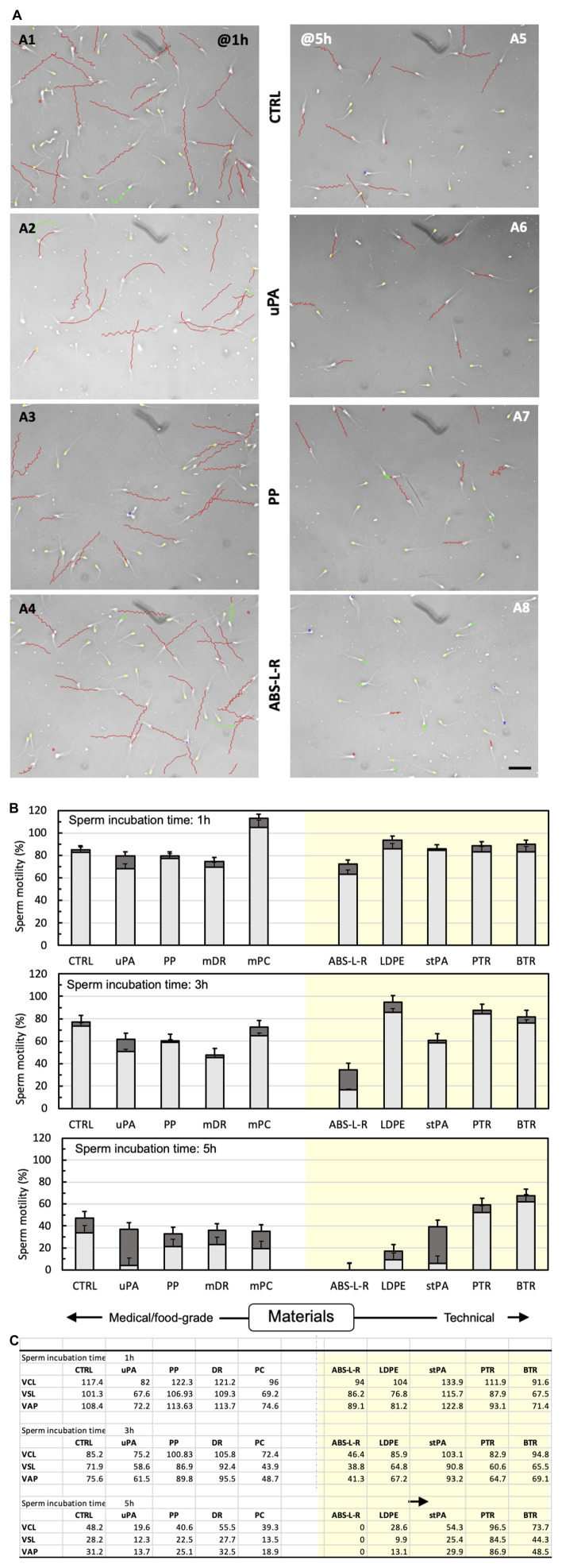
Cytocompatibility of bioreactor material candidates according to the quick sperm test. Material cytocompatibility was characterized in terms of the effect on spermatozoa motility and kinetic parameters of leachables extracted with the medium for 72 h from various materials (*n* = 2–3 depending on availability). **(A)** Computer-assisted analysis of sperm motility after 1 h (A1–A4) or 5 h (A5–A8) incubation with leachable-containing medium from selected materials: A1, A5—control medium; A2, A6—uPA; A3, A7—PP; A4, A8—ABS-L-R. Images show sperm tracks in the elapsed time, colour-coded to indicate the estimated sperm motility: red, rapid progressive motility (type a); green, slow progressive motility (type b); blue, non-progressive motility (type c); yellow, immotile. Bar = 100 μm; elapsed time, Δt = 1 s. **(B)** Spermatozoa motility: total motility (whole bar) and motility of progressive (light grey) and non-progressive (dark grey) spermatozoa incubated for 1 h, 3 h, or 5 h with control medium (CTRL) or leachable-containing medium from food/medical-grade (left clear panel) and technical materials (right-shaded panel). **(C)** Kinetic parameters: curvilinear velocity (VCL), straight line velocity (VSL), and average path velocity (VAP) of spermatozoa incubated for 1 h, 3 h, or 5 h with control medium (CTRL) or in leachable-containing medium from food/medical-grade (left clear panel) or technical materials (right shaded panel). Experiments were performed in triplicate.

The prototypes in the final configuration were manufactured to gather information on the effectiveness of tissue culture in the novel perifusion bioreactor. The materials used were selected on the basis of their cytocompatibility, quickly assessed by characterizing the effect of leachables on the motility of bull sperms. The material cytocompatibility predicted by the quick test was eventually validated in long-term culture experiments with ovarian tissue in the perifusion bioreactor. Figures 8A–C show that the leachables released in the medium generally worsened the progressive motility and motion kinetic parameters of the spermatozoa, to a greater extent the longer the incubation time with spermatozoa and the poorer the material cytocompatibility (i.e., sperm compatibility). Accounting for the percent of non-progressive spermatozoa, the BTR and PTR technical resins affected spermatozoa motility the least at all incubation times, followed by food-grade PP, medical-grade PC, and dental resin DR. The technical BTR and PTR had effects on the sperms that were similar to, or even better than, culture dish polystyrene, sperms exhibiting a 55% better motility with leachables from BTR and PTR than the control after 5 h of incubation. Medical-grade uPA and technical stPA worsened spermatozoa motility only after 5 h of incubation, eventually yielding the greatest fraction of non-progressive spermatozoa among the tested materials (i.e., from 2% to 15% after 1–3 h incubation to 87% on average). ABS-L-R was the least cytocompatible material and worsened spermatozoa motility to the greatest extent, the total motility decreasing from 85% of the control, to 45%, to 0% after 1 h, 3 h, and 5 h incubation, respectively. A detailed statistical comparison of the cytocompatibility of material pairs is provided in Supplementary Table S1.

### 3.4 Tissue culture in perifusion bioreactor prototypes

**FIGURE 9:**
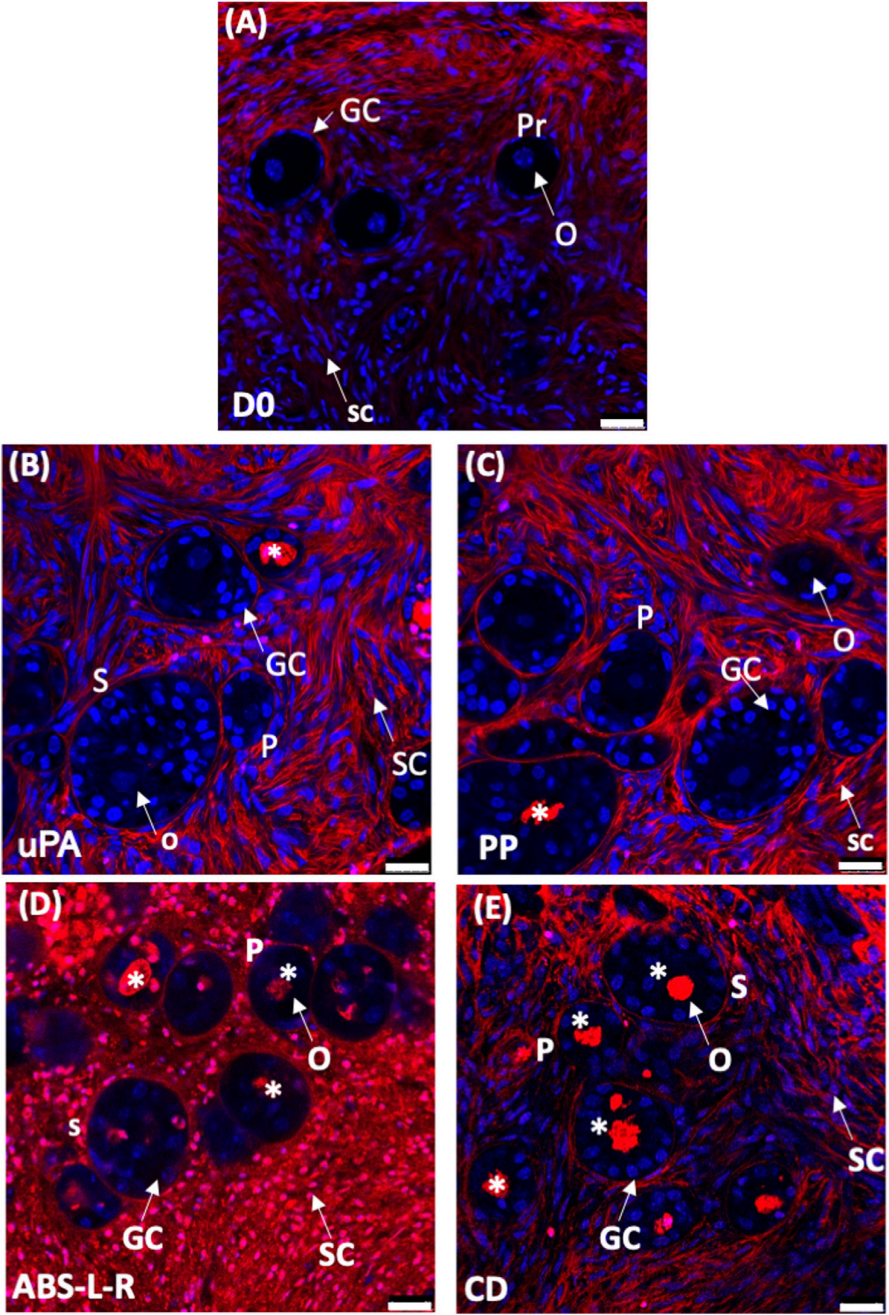
Viability of ovarian tissue cultured in different bioreactors. Representative confocal images of LIVE/DEAD staining of ovarian tissue: **(A)**, fresh @D0; dynamically cultured in perifusion bioreactors made of different materials: **(B)**, untreated polyamide, uPA-PB; **(C)**, polypropylene, PP-PB; **(D)**, ABS-like resin, ABS-L-PB; **(E)**, in static culture in static dish culture, CD. Images show: in blue, nuclei stained with Hoechst 33342; in red, extracellular matrix and dead cells stained with LIVE/DEAD Far Red. Symbols: *, dead follicles; GC, granulosa cells; O, oocyte; Pr, primordial follicle; P, primary follicle; S, secondary follicle; SC, stromal cell. Bar = 25 μm.

**FIGURE 10:**
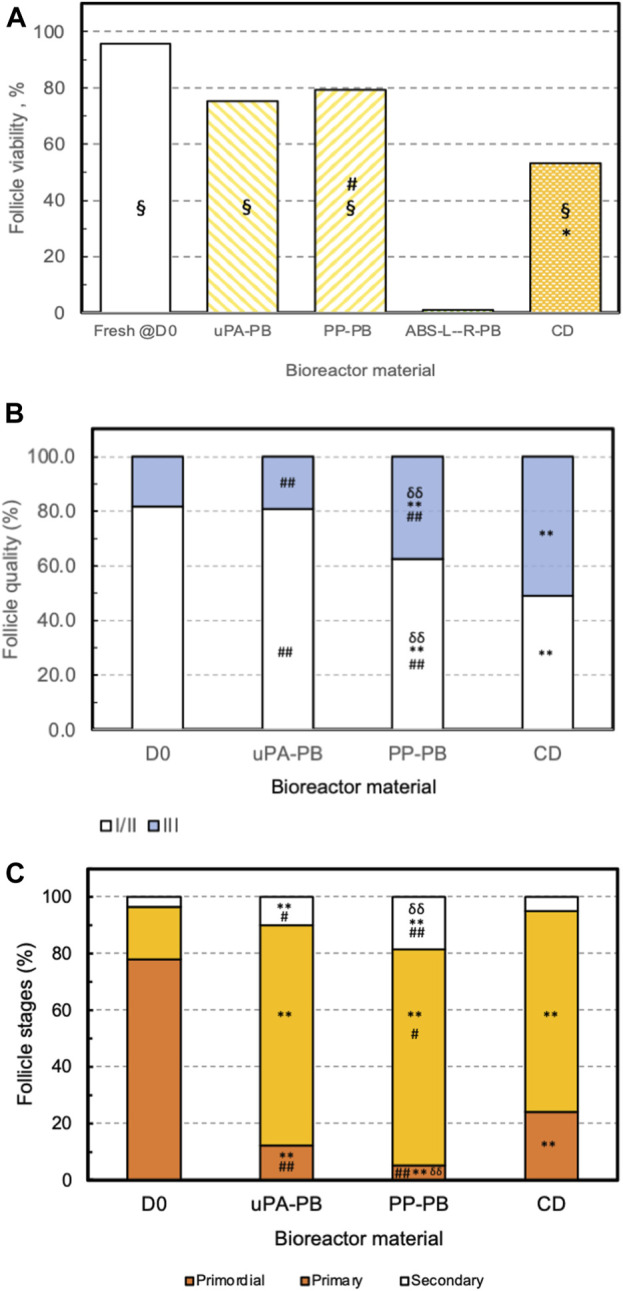
Effectiveness of long-term ovarian tissue culture in perifusion bioreactors made of different materials. Effectiveness of follicle *in situ* culture in bioreactors made of various polymeric material (uPA-PB, *n* = 1; PP-PB, *n* = 3; ABS-L-R-PB, *n* = 1) as compared to fresh tissue @D0 (D0) and conventional dish culture (CD) (please see text for acronyms). Effectiveness estimated from the histological characterization of fresh and cultured ovarian cortical tissue in terms of **(A)** follicle viability. Statistically significant differences reported as *, CD vs. D0; **§**, uPA-PB and PP-PB vs. CD vs. ABS-L-R-PB; **#**, PP-PB vs. CD. **(B)** Follicle health or grading. Statistically significant differences reported as **, *p* < 0.01, D0 vs. PP-PB and CD; **##**, *p* < 0.01 CD vs. uPA-PB and PP-PB; **δδ**, *p* < 0.01, PP-PB vs. uPA-PB. **(C)** Follicle progression or staging. Statistically significant differences reported as **, *p* < 0.01, D0 vs. uPA-PB, PP-PB, and CD; **#**, *p* < 0.05, CD vs. PP-PB and uPA-PB; **##**, *p* < 0.01, CD vs. uPA-PB and PP-PB; **δδ**, *p* < 0.01, PP-PB vs. PA-PB (please read text for acronyms).

Based on the outcome of the cytocompatibility test, the prototypes of the novel perifusion bioreactor were used for the preliminary culture experiments which were made of PP (i.e., a cytocompatible material according to the sperm test) (PP-PB); uPA (i.e., a material poorly cytocompatible only long-term) (uPA-PB); and ABS-L-R (i.e., the least cytocompatible material) (ABS-L-R-PB). Representative confocal images of tissue cultured for 7 days in perifusion bioreactors and in static culture dish are shown in Figure 9. They show the presence of viable small-sized primordial follicles in fresh tissue @D0 (Figure 9A) and that most follicles in tissues cultured in uPA-PB and PP-PB were viable after 7 days of culture (Figures 9B, C). Figure 9D shows that in tissues cultured in ABS-L-R-PB for 7 days very few follicles were still viable and also the surrounding stromal cells were non-viable. Figure 9E shows that after 7 days of static culture, most follicles were non-viable but they were surrounded by viable stromal cells. After 7 days in *in situ* culture, the quantitative characterization of follicles (*n* = 956 total follicles of which D0 = 211 and @D7 = uPA-PB, 339; PP-PB, 227; ABS-like-R-PB, 53; CD, 126) showed that the percent of viable follicles in PP-PB or uPA-PB was significantly higher than in CD (i.e., 78% and 75% vs., 53%, respectively) and only slightly lower than in fresh D0 tissue (i.e., 96%) (Figure 10A).

**FIGURE 11:**
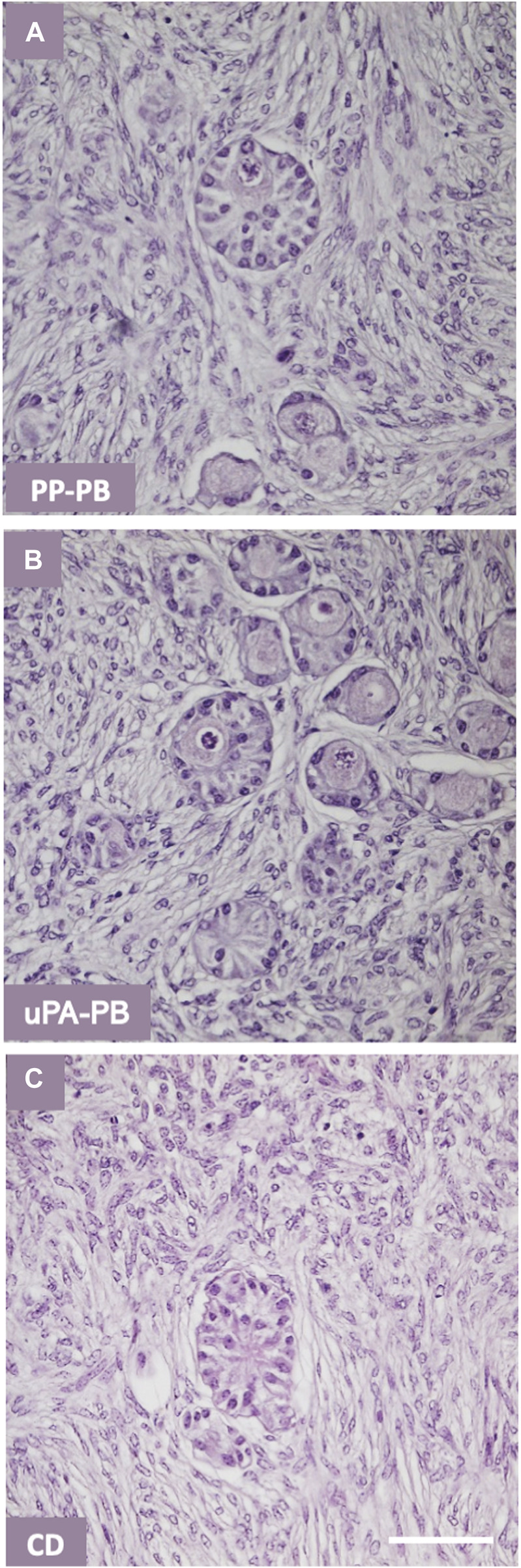
Quality of secondary follicles in ovarian tissue cultured in various bioreactors. Representative histology of secondary follicles in ovarian tissue cultured for 7 days in various bioreactors: **(A)** dynamic culture in PP-PB; **(B)** dynamic culture in uPA-PB; **(C)** static culture in conventional dish, CD. Bar = 100 μm.

The histological analysis allowed to characterize follicle quality (i.e., grading) and development (i.e., staging) in tissues cultured in uPA-PB, PP-PB, and CD in comparison with fresh D0 tissues (n = 2,060 total follicles of which D0 = 689 and @D7 = uPA-PB, 178; PP-PB, 670; CD, 523). The histological analysis of tissue cultured in ABS-L-R-PB was not performed for the great loss of viable follicles @D7. Figure 10B shows that follicles in the ovarian strips cultured in the novel dynamic perifusion bioreactors were generally healthier than in the static culture dish with a significantly greater fraction of healthy (i.e., grade I and II) follicles. The percent of healthy follicles in tissue cultured in uPA-PB @D7 was significantly higher than in PP-PB (80.9% vs. 62.5%, respectively) and was similar to fresh D0 tissue (81.9%). Correspondingly, the fraction of atretic (i.e., grade III) follicles was significantly higher in CD than in PP-PB, uPA-PB, and fresh D0 tissue (i.e., 51.1%, 37.5%, 19.1% and 18.1%, respectively). Figure 10C shows that after 7 days of culture, the fraction of primordial follicles generally decreased with a simultaneous significant increase in the fraction of primary follicles with respect to fresh D0 tissue. The fraction of secondary follicles in tissues cultured in the novel perifusion bioreactors made of either material was significantly greater than in the static culture dish, being 18.7%, 10.1%, and 5.0% in PP-PB, uPA-PB and CD, respectively. Figure 11 shows that healthy stromal cells surround the secondary follicles produced after 7 days of dynamic culture in PP-PB (Figure 11A) and uPA-PB (Figure 11B). Figure 11C shows a typical atretic secondary follicle surrounded by loose ECM after 7 days in static culture.

## 4 Discussion

*Folliculogenesis* is a highly complex biological process involving oocytes, somatic cells of the follicular and stromal compartment, as well as the ECM. This process also depends on their time- and stage-dependent interactions via endocrine, paracrine, and autocrine biochemical signals (Eppig, 2018) and mechanotransduction processes (Martinez et al., 2021). The goal of this study was to develop a bioreactor that generates an *in vitro* microenvironment fostering ovarian follicle health and development and overcomes the limits of conventional culture dishes. The current incomplete understanding of ovarian *folliculogenesis*, particularly in humans (Hoffmann et al., 2012), makes bioreactor development particularly difficult. Culturing follicles *in situ* permits the exchange of biochemical signals among ovarian cells occurring inside small strips of intact ovarian cortex (Yang et al., 2020). The hypothetical rationale to develop the novel bioreactor design was gathered by analysing the structural/mechanistic aspects of follicle maturation *in vivo* and the *in vitro* experimental approaches reported in the literature. Particular attention was paid to possible synergies between biochemical and biomechanical signals. Both structural aspects and the literature hinted at the importance during follicle development of the regulation of dissolved oxygen and of mechanotransduction (Kehoe et al., 2021; Matsuzaki, 2021).

In fact, the ovarian follicle is a highly specialized tissue that metabolically requires oxygen at concentrations and in amounts that vary as it undergoes complex structural changes and travels in the ovary from the poorly vascularized cortex to the medulla during its development to the antral stage (Fischer et al., 1992). *In vivo*, hypoxia is thought to play a role in keeping primordial follicles quiescent (Makanji et al., 2014). *In vitro*, hypoxic culture conditions have been reported to limit the development of bovine follicles (Talevi et al., 2018; Ma et al., 2019) by inhibiting oestrogen receptor α (Ma et al., 2019), maintain oocytes in the quiescent state in ovaries engineered from pluripotent stem cells, via the overexpression of forkhead box O3 (FOXO3) through hypoxia-inducible factors (John et al., 2008; Shimamoto et al., 2019), and lower the reserve of primordial follicles in rats (Aiken et al., 2019). The enhancement of oxygen transport to tissue has been shown to enhance development and health of bovine (Talevi et al., 2018; Catapano et al., 2019) and mouse (Connolly et al., 2019) follicles, apparently as long as the dissolved oxygen concentration is within an optimal range (Talevi et al., 2018).

The potential importance of mechanotransduction to follicle activation and development may be inferred from studies investigating the effects of stretching on monolayers of densely packed MDCK cells and of *Drosophila* follicle cells (Benham-Pyle et al., 2015; Fletcher et al., 2018). In brief, in the absence of tensile stimulation, the pressure acting on MDCK cells as an effect of the dense cellular packing keeps them quiescent by inhibiting the transcription coactivator yes-associated protein (YAP) via the Hippo pathway (Zhao et al., 2007; Benham-Pyle et al., 2015). The application of tensile biaxial solid mechanical strains reportedly stretches and flattens the cells, loosens their contacts in the monolayer and relaxes pressure, and ultimately induces a rapid cell cycle re-entry and cell proliferation mediated by the overexpression of Yap1 and β-catenin transcriptional activities and nuclear re-localization of cytoplasmic YAP, although with different response times and durations (Benham-Pyle et al., 2015). Similarly, Fletcher et al. have shown that stretching follicular cells of *Drosophila* makes the cells change shape and flatten, reduces apical Hippo dimerization, and causes Yki (i.e., Yorkie, the *Drosophila* homolog of YAP) gene activation and translocation into the cell nucleus that ultimately increases cell proliferation (Fletcher et al., 2018). Such activation has been reported to be independent of the cell type (Fletcher et al., 2018). Interestingly, Telfer and McLaughlin (2017) have also reported that challenging strips of ovarian cortical tissue of large mammals with a short (at least) 10% uniaxial tensile strain enhances health and viability of follicles that develop well in subsequent *in vitro* culture. Based on such findings, the novel bioreactor was conceived to enable the effective transport to tissue of medium-borne dissolved oxygen, and an adequate mechanical stimulation of the tissue strips.

### 4.1 Transport enhancement

*In silico* models of flow and dissolved oxygen transport were used throughout to explore the effectiveness with which various bioreactor configurations enhance oxygen transport to tissues, being aware of the limitations associated with the approximations in the mathematical description of the bioreactor components and in parameter estimation. Once the tissue is disconnected from the ovarian vasculature and cultured *in vitro*, oxygen and nutrients have to be supplied to the ovarian cells through the strip's outer surfaces where solutes are carried with the medium. Different from the culture of engineered cellular constructs, at least soon after cell seeding, the high hydraulic resistance of the ovarian tissue does not permit the medium to flow around the cells inside the tissue (i.e., to perfuse tissue with medium) (Devireddy, 2005; Fernandez et al., 2013; Rohanifar et al., 2022). For this reason, we focused on the design of a perifusion bioreactor, in which the medium flows outside and around the tissue strips carrying dissolved oxygen to the tissue that eventually diffuses to the cells across the tissue strip.

The first main design objective was to maximize oxygen transport from the bulk medium to the tissue surface. To ensure reproducible performance, the bioreactor design (i.e., its configuration, geometry, and operating conditions) had to ensure that the tissue strips are in fixed space positions, at a convenient and regular distance from one another; the strip's surface area exchanging solutes with the medium is maximal; and the resistance to dissolved oxygen transport from the bulk medium to the follicles inside the tissue is minimal. The high diffusional resistance to solute transport caused by the dense ovarian stroma and the high density and metabolic requirements of ovarian cells (i.e., stromal and follicular cells) (Fraser et al., 1973; Haslene-Hox et al., 2015) force to keep the strip size small to supply oxygen and nutrients to the cells anywhere inside the strip and maintain them viable after *in vitro* follicle development and strip implantation in the host (Ding et al., 2010). The small size hindered the possibility of using usual clamps and actuators to grasp the strips, keep them in fixed space positions, and subject them to mechanical *stimuli*. In fact, tools generally used for this purpose are big (as compared to the strips) and made of gas-impermeable material. Their use would blind the strip solute exchange surface area and cripple oxygen transport to the ovarian cells. Sandwiching the tissue strips between open-mesh nets with a mesh size small enough to retain the strips, yet large enough to enable their perifusion, effectively maintained the strips in a fixed space position and enabled the application of direct mechanical strain on tissue. Their use also facilitates the strips' loading into and retrieval from the bioreactor.

Perifusing the strips with the medium makes it possible to control oxygen supply to the tissues and tissue stimulation with fluid mechanical shear stresses by changing the inlet medium flow rate. The 3D velocity profiles predicted by the flow model as bioreactor configuration evolved provided information on the extent to which strip perifusion was enhanced. For the sake of the example, model predictions suggested that positioning the strips close to one another at the centre of the porous support would maximize strip perifusion and ensure fluid dynamics stimulation at the given medium inlet flow rate (Figure 3). Model predictions suggested also that oxygen transport to tissue could be further enhanced by facilitating medium flow underneath the strips. The maximal enhancement of oxygen supply to cells was obtained by carving (or 3D-printing) communicating concentric channels on the upper surface of the porous support, to promote medium flow below the strips before leaving the culture chamber, and by interposing an open-mesh net between the strip bottom and porous support, to facilitate the lateral flow of medium parallel to the porous support surface (Figure 4). These features increased the strip surface area bathed by the medium and the velocity at which the medium flows along, close to the strip's surfaces. This added some degree of convection to an otherwise purely diffusive oxygen transport to the tissue and enhanced its fluid dynamic stimulation. For a given oxygen concentration difference between the medium bulk and tissue–medium interface, the rate at which oxygen is transported to the tissue may be assumed proportional to the strip's mass exchange surface area and inversely proportional to the external resistance to oxygen transport. According to the “film model,” the resistance to oxygen transport is concentrated in the stagnant layer of the medium adhering on the strip surface (Bird et al., 2002). The 3D distribution of dissolved oxygen concentration predicted by the oxygen transport model for the final bioreactor operated at *J*_*F*_ = 4.8^∙^10^–5^ m/s confirmed that increasing the strip's mass exchange surface area and medium mixing close to the strips may effectively enhance oxygen transport between the bulk medium and strip over conventional dishes (Figure 5). In fact, Figures 5A–D show that the tissue strips are cultured at oxygen concentrations above the 1%–2% threshold, below which oocytes' viability may be compromised (Harris and Picton, 2006). The extent of oxygen transport enhancement may be clearly appreciated in Figures 5E, F, which show that the thickness of the model-predicted stagnant layer across which oxygen is transported (hence, the oxygen transport resistance) above an exemplary tissue strip is about an order of magnitude lower in the perifusion bioreactor than it is in the static culture dish.

To explore whether bioreactor performance could be better exploited by varying the perifusing superficial flux *J*_*F*_, *in silico* experiments were performed to predict bioreactor behaviour over a wide range of *J*_*F*_. Figure 6 shows that operating the perifusion bioreactor at increasing *J*_*F*_ enhances oxygen transport and oxygen concentration at the strip's surface; the higher the *J*_*F*_, the more the oxygen at the strip's surface. The associated changes of the dimensionless groups *ϕ*_*oxy*_ and *Bi*_*m,oxy*_ help better understand to what extent varying the *J*_*F*_ affects the conditions under which the ovarian cells are cultured inside the strips. In fact, *ϕ*_*oxy*_ >> 1 suggests that high oxygen cellular consumption, or poor diffusion, limits oxygen transport inside the tissue. *Bi*_*m,oxy*_>> 1 suggests that diffusion inside the tissue limits oxygen supply to ovarian cells. At low *J*_*F*_, it is approximately *Bi*_*m,oxy*_ ∼1. Together with the high strip *ϕ*_*oxy*_^2^∼12, the parameter values suggest that oxygen transport resistances both external to and inside the tissue strips contribute to limiting oxygen supply to cells and cause significant oxygen concentration drops outside and inside the tissue strip. Increasing *J*_*F*_ increases *Bi*_*m,oxy*_, whereas *ϕ*_*oxy*_^2^ remains constant. At the highest *J*_*F*_ considered, *Bi*_*m,oxy*_∼18 suggests that the external resistance to oxygen transport has been minimized (hence the oxygen concentration drop mostly occurs inside the tissue strip) and that oxygen supply to cells inside the strip is only limited by the transport and metabolic properties inside the tissue. This suggests operating the perifusion bioreactor at the highest possible superficial filtrate flux *J*_*F*_. However, Figure 5 shows that increasing *J*_*F*_ causes tissue stimulation with fluid dynamic shear stresses that increase from about 10^–4^ Pa to 10^–1^ Pa. Tissues can benefit from stimulation at shear stresses, provided they are in the 10^–3^–10^–2^ Pa range. In fact, shear stresses in this range are physiological for many cells, such as proximal tubule cells (Raghavan et al., 2014) and intestinal epithelial cells (Kim et al., 2012), and have been reported to elicit the biochemical response of many cells *in vitro* (Wang and Tarbell, 2000; McCoy and O’Brien, 2010; Kim et al., 2014; Esch et al., 2015). However, culture at shear stresses as high as 10^–1^ Pa might damage tissue. In fact, shear stresses this high have been reported to negatively affect mesenchymal adipose-derived stem cells (Kaiser et al., 2013) and CHO cells (Keane et al., 2003). Moreover, the effects of shear stress on cells are often characterized in experiments that last only from a few minutes to a few hours, whereas the ovarian tissue is kept in culture for weeks. For these reasons, it was decided to operate the perifusion bioreactor at *J*_*F*_ = 4.8^.^10^–5^ m/s to exploit the enhanced oxygen transport and physiological fluid mechanic stimulation that could be obtained while minimizing possible metabolic damages to the tissue. In fact, at *J*_*F*_ = 4.8^∙^10^–5^ m/s, the oxygen concentration at the strip's surface is already well above the hypoxic threshold, and further flux increases are not metabolically relevant, and the shear stresses acting on the strips would not exceed the 10^–2^ Pa threshold.

### 4.2 Direct mechanical stimulation

The second main design objective was to enable the direct biomechanical stimulation of the tissue to exploit possible interactions between the biochemical and biomechanical *stimuli*. Starting from the most transport-effective bioreactor configuration, the height of the ribs protruding from the vault of the bioreactor inlet half and the thickness of the two open-mesh nets enveloping the strips was slightly adjusted so that direct solid compressive strains were exerted on the tissue strips. The nets served also to distribute over the whole strips the solid compressive strains locally exerted by the ribs and net components. The final bioreactor geometry permitted to challenge the strips with solid strains, the depth of which would likely elicit the biochemical response of the ovarian tissue without damaging it. To overcome the scarce quantitative knowledge and the wide variability of the mechanical behaviour of ovarian tissue (Pascoletti et al., 2020; Stewart et al., 2023) and nets, direct mechanical stimulation was characterized in terms of the depth of the strains exerted on the tissue in the strip-loading experiments in which the tissue was replaced with wax mock strips, with dominant plastic behaviour. The strain depth was measured by 3D profilometry analysis. Figure 7 shows that strains were non-uniformly distributed on the strip surface and featured depth and shape that depended on whether the knots or filaments of the net were in direct contact with the mock strip. The maximal total strain exerted on the strip surface was found to range up to a maximum of about −15%. Strains this deep are close to those that reportedly stimulate the mechanobiological response of ovarian cortical tissue (Telfer and McLaughlin, 2017), and of many cells, such as embryonic stem cells (Shimizu et al., 2008), dermal fibroblasts (Xu et al., 2017), and epithelial pulmonary cells (Chaturvedi et al., 2007). On average, they are also below the 12%–33% tensile strain threshold associated with failure of soft tissues (e.g., the tendons, brain, liver, kidneys, or spleen) (Singh and Chanda, 2021) and the −45% compressive strain threshold for the failure of the muscle tissue (Ceelen et al., 2008).

A word of caution is in order on the technique used to characterize the strain exerted on the tissue during culture in the perifusion bioreactor. In fact, the technique actually measures the depth of the residual plastic deformation left by the nets on the wax mock strip after this is recovered from the bioreactor. Such deformation depends on the strip thickness and the viscoelastic behaviour of the tested material. To minimize possible bias, a tissue cutter was developed that reliably delivers tissue strips of 600-μm thickness (Barbato et al., 2023). Accounting for the viscoelastic behaviour of the tissue and nets would have required a thorough characterization of the ovarian tissue's and nets' mechanical behaviour and the development and validation of a suitable mechanical model of the bioreactor set-up, which is beyond the scope of this work. For practical reasons, it was preferred to apply the reported characterization technique to mock strips made of the Cliadent wax used for braces. This specific wax is preferred to other mock materials because many of those available would stick to the nets, would not hold the strip's shape, or would exhibit a dominant elastic behaviour. However, possible differences in the viscoelastic behaviour of the ovarian tissue and the mock wax may indeed bias the strain estimates that have been obtained. Another source of bias is the wide variations in tissue structure (van Wezel and Rodgers, 1996; Grosbois et al., 2023) and mechanical behaviour from tissue donor to donor and from different harvesting zones of the same ovary. For the sake of an example, the Young’s modulus of the ovarian cortex has been reported to vary by as much as two orders of magnitude in different zones of the same ovary (Stewart et al., 2023).

### 4.3 Cytocompatibility of material candidates

Predicting, on purely theoretical grounds, how follicles would develop in the culture's microenvironment that is established in the novel perifusion bioreactor is hindered by the current incomplete quantitative knowledge of ovarian tissue metabolism. For this reason, prototypes in the final configuration were manufactured and used for characterizing the effectiveness of ovarian tissue culture in the novel perifusion bioreactor. In cell bioreactor design, the effects on the cultured tissue of the materials used are often underestimated, or even neglected. In tissue engineering, bioreactors are generally made of certified medical-grade polymers by subtractive manufacturing techniques or by soft-photolithography. 3D-printing additive techniques are becoming more and more attractive because they enable the quick and economic manufacturing of bioreactor prototypes of complex geometry using many polymeric materials. Provided that they are certified cytocompatible by regulatory bodies (e.g., FDA, ISO, etc.), materials for 3D-printing cell bioreactors are generally selected on the basis of their cost and accuracy permitted by the associated manufacturing technique. Unfortunately, chemicals are often added to the polymer dope used for printing as plasticizers, photoinitiators, or light stabilizers. Reproductive cells (e.g., ovarian tissue cells, follicles, and spermatozoa) are also more sensitive to toxicants than other cells or tissues, some of which are used to certify material biocompatibility (ISO, 2009). Recently, Rogers and colleagues proved that bioreactors for mouse oocyte culture that were 3D-printed with some commercial medical-grade dental resins are ovotoxic for the presence of chemicals (e.g., Tinuvin 292) that may be released in the culture medium if not washed away or thoroughly reacted (Rogers et al., 2021). On these grounds, we purposely developed a quick and economic test to screen indirect cytotoxicity towards the reproductive tissue of material candidates for the novel bioreactor. The test characterizes the effect on the motility of bull spermatozoa of prolonged contact with leachables extracted from the materials of interest. Surprisingly, but consistent with that reported in Rogers et al. (2021), some technical materials proved less cytotoxic than some medical-grade materials, the latter apparently releasing more spermotoxic chemicals (Figure 8). More detailed information on the statistical significance of cytocompatibility differences between material candidates for bioreactor construction is available in Supplementary Table S1. Long-term culture experiments with bovine ovarian strips in perifusion bioreactors manufactured with materials of select sperm-compatibility yielded information on the effect of these materials on follicle viability that is consistent with those obtained with spermatozoa. This confirmed that the quick sperm test predicts material cytocompatibility with ovarian tissue well (Figures 8, 10).

### 4.4 Tissue culture in perifusion bioreactor prototypes

To characterize the effectiveness of ovarian cortical tissue strips cultured in the novel perifusion bioreactors, as well as the influence of material cytotoxicity on bioreactor performance, prototypes were manufactured for the culture experiments with polypropylene (PP), a biomaterial of long-term cytocompatibility; untreated polyamide (uPA), a material of short-term cytocompatible but toxic in long term; and the cytotoxic ABS-like resin (ABS-L-R). The outcome of the experiments with perifusion bioreactors made of polypropylene (PP-PB) and untreated polyamide (uPA-PB) suggests that culture in the novel dynamic bioreactor permits to overcome many obstacles hindering the successful long-term culture of small-sized strips of ovarian cortical tissue in static culture. In fact, the culture in perifusion bioreactors made of either material promoted the development of a significantly greater number of primordial follicles to the secondary stage and yielded follicles that were more viable and in better health, than static culture in a conventional dish (Figures 9–11). Such results are likely caused by the remarkable enhancement of oxygen transport between the bulk medium and tissue effected by the maximization of the mass exchange surface area of the tissue strips and the minimization of oxygen transport resistance external to the strips (Figures 4, 5) achieved in the final bioreactor design.

The steady mechanical stimulation to which the tissue is subjected in the perifusion bioreactor and that is missing in static culture dishes may have also contributed to the results that were obtained. In fact, the compressive strains exerted orthogonal to the top and bottom surfaces of the soft ovarian tissue strips may have triggered a squeeze flow in the other unconstrained directions (Meeten, 2002) that steadily stretched the follicular cells. Consistent with that reported in Benham-Pyle et al. (2015) and Fletcher et al. (2018), such stretching may have contributed to triggering follicle activation and promoting the follicular development and viability observed in the perifusion bioreactor culture. It is difficult to estimate the extent to which tissue mechanical stimulation influences the observed activation and development of the bovine ovarian follicles. Some similarity exists between that reported in MDCK cells and *Drosophila* follicular cells (Benham-Pyle et al., 2015; Fletcher et al., 2018) and that reported on the metabolic effects of ovarian tissue fragmentation or disruption used since 1930 for inducing follicular development (Stein and Leventhal, 1935; Donnez et al., 2011; Farquhar et al., 2012). This evidence hints at a role of mechanotransduction. In fact, similar to the effect of the dense cellular packing on MDCK cells (Benham-Pyle et al., 2015), the pressure exerted by the granulosa cells and the stiff ECM of the ovarian cortex on the oocytes keeps murine primordial follicles quiescent. Loosening the ECM by digestion with collagenase relaxes pressure and activates the oocytes, as a result of the nuclear translocation of FOXO3 (Nagamatsu et al., 2019), similar to that occurring upon stretching the tissue. Moreover, follicle activation and development promoted by the fragmentation of murine ovaries followed by strips' autotransplantation has been shown to occur along similar pathways to those through which *Drosophila* follicular cells are activated by stretching, such as the disruption of the Hippo-signalling pathway and the activation and nuclear translocation of YAP (or the homolog Yorkie in the case of *Drosophila* cells) (Kawamura et al., 2013; Fabregues et al., 2018; Ferreri et al., 2020; Hsueh and Kawamura, 2020; Kawamura et al., 2020). A hint at the importance of the direct mechanical stimulation is provided by the failure of an exploratory dynamic culture experiment performed with a stainless-steel bioreactor in the absence of direct mechanical stimulation (Table 2). Table 2 shows that after 7 days of culture of 10 tissue strips, sandwiched between two meshes and laid on a uniformly porous support, in the absence of direct mechanical stimulation at conditions similar to the experiments with the final bioreactor configuration, not a single follicle survived and the tissue was healthier in the static culture. Investigating the extent to which oxygen transport enhancement, fluid dynamic mechanical stimulation, and direct mechanical stimulation separately influence follicle activation, development, and viability would require the development of specific experimental setups and a specific investigation, which is beyond the scope of this work.

**TABLE 2:** Effectiveness of strip culture in the dynamic bioreactor portrayed in Figure 1B. Outcome of an exploratory culture experiment with strips loaded in an exemplary stainless-steel perifusion bioreactor (PB) where the strips were laid on a uniformly porous support sandwiched between two open-mesh nets and cultured without mechanical stimulation (Figure 1B). 2D static culture in dishes with a gas-permeable bottom (PD) served as the control, as shown in Talevi et al. (2018). Culture effectiveness estimated in terms of **(A)** follicle health or grading; **(B)** follicle progression or staging.

Panel (A)	Grade 1, %	Grade 2, %	Grade 3, %
@D0	42.7	20.8	36.5
PD, @D6	44.8	15.2	40
PB, @D6	0	0	100

Panel (B)	Primordial, %	Primary, %	Secondary, %
@D0	91.6	2.8	5.6
PD, @D6	45.7	44.8	9.5
PB, @D6	NC	NC	NC

Culture conditions for PB and PD were the same as that for PB and static culture in conventional dish (CD) experiments, respectively. NC, not classified.

The comparison of the culture's outcome with perifusion bioreactors made of materials that are spermotoxic to different extents (Figures 8, 10) shows that material cytocompatibility for ovarian tissue strongly conditioned bioreactor performance and culture effectiveness. After 7 days of culture, neither healthy nor viable follicles could be found in the ovarian tissue cultured in perifusion bioreactors made of the cytotoxic ABS-like resin. In the tissue cultured in perifusion bioreactors made of the more biocompatible untreated polyamide, follicles were healthier and progressed to the secondary stage to a greater extent than in the static culture. Follicle viability and the fraction of secondary follicles in the tissue cultured in perifusion bioreactors made of polypropylene were significantly higher than in bioreactors made of untreated polyamide, with follicle progression to the secondary stage also being thrice higher than in the static culture. This outcome may likely be attributed to better cytocompatibility in the long term with ovarian tissue of polypropylene than of untreated polyamide. It is noteworthy that the long-term culture of cortical ovarian tissue was more effective in perifusion bioreactors made of materials that proved more cytocompatible according to the quick test based on the use of bull spermatozoa developed in this work. This confirms that the test reliably and quickly predicts the cytocompatibility of materials also with ovarian tissue.

Taken together, the experimental results support the greater effectiveness of culture in the novel dynamic perifusion bioreactor than in static culture. The remarkable enhancement of follicle viability, health, and development obtained by culturing ovarian tissue in perifusion bioreactors over static dish culture highlights the importance for follicle development and health of recreating *in vitro* the synergistic effects between applied biochemical and biomechanical signals. The results also stress the importance of a careful material selection driven by purposely developed cytocompatibility tests for bioreactors in which reproductive tissue is cultured long term. Gaining a better understanding of ovarian tissue metabolism and its interactions with challenges of the external environment is of paramount importance. This new knowledge may help develop bioreactors in which the tissue is cultured in a more physiological and controlled environment than the one herein proposed and that may effectively be used for carrying out a full *in vitro folliculogenesis*.

## 5 Conclusion

In this study, we report the development of a novel dynamic perifusion bioreactor designed to enhance the effectiveness of ovarian tissue strip culture over static dish culture by exploiting the synergic ovarian tissue response to biochemical and biomechanical *stimuli*. The outcome of preliminary culture experiments with fresh bovine tissue suggests that in the novel bioreactor, the enhancement of dissolved oxygen transport from the medium to the tissue coupled to the biomechanical stimulation of the tissue with steady solid compressive strains and fluid dynamic shear stresses yields a significant enhancement of primordial follicle activation and development into secondary follicles, when compared to the results achieved using the static dish culture. This provides proof of concept that culture in the novel bioreactor overcomes the impediments of conventional dish culture. A custom-made quick test was developed using bull sperms to assess the cytocompatibility with ovarian tissue of material candidates for bioreactor construction. The test reliably also predicted material cytocompatibility with ovarian tissue. Long-term ovarian tissue culture in the novel bioreactor significantly worsened when bioreactor prototypes were manufactured with cytotoxic materials, irrespective of whether some of them are certified cytocompatible, according to current regulations. This strengthens the importance of suitable materials' cytocompatibility assessment for reproductive tissue culture.

## Figures and Tables

**Table:** 

**Bi**_**m,oxy**_ **= k**_**c,oxy**_ * *δ*_**tiss**_ **/D**_**oxy,tiss**_	Biot number for oxygen, medium bulk to tissue transport, —
**C**_**oxy,in**_	Oxygen concentration in inlet stream, mol/m^3^
**D**_**oxy,j**_	Oxygen diffusivity in the *j*-th compartment, m^2^/s
**d**_**tubing**_	Inlet/outlet tubing diameter, m
**J**_**F**_	Superficial filtrate flux, m/s
**K**_**M,oxy**_	Michaelis constant for oxygen consumption, mol/m^3^
**k**_**c,oxy**_ **=D**_**oxy,m**_ **/*δ***_**sl**_	Oxygen transport coefficient at medium-tissue interface, m/s
**k**_**net**_	Darcy permeability of the nets, m^2^
**k**_**PS**_	Darcy permeability of the porous support, if applicable, m^2^
**R**_**in**_	Radius of the inlet tube, m
**S1**	Fractional total strip top surface area exposed to shear stresses between 10^−3^ Pa and 10^−2^ Pa, %
**S2**	Fractional total strip top surface area exposed to medium velocity greater than 1.8∙10^−3^ m/s, %
**V**_**max,oxy**_	Maximal oxygen consumption rate of cortical tissue, mol/(s∙m_tiss_^3^)
**x =(x, y, z)**	Vector of space coordinates
**z**	Axial coordinate [m]
**Greek symbols**	
***δ***_**sl**_	Thickness of the medium stagnant layer adherent on tissue strip, m
***δ***_**SS**_	Tissue strip spacing, m
***δ***_**tiss**_	Tissue strip thickness, m
***ε***	Porosity, —
***μ***	Medium viscosity, Pa∙s
_**ρ**_	Medium density, kg/m^3^
***ϕ* =√(V**_**max,oxy**_ ***δ***_**tiss**_ **/(D**_**oxy,tiss**_ **C** ^**oxy,in**^ **)**	Oxygen Thiele modulus in a strip, —
**Superscripts and subscripts**	
**in**	Inlet
**max**	Maximal
**m**	Medium
**net**	Nets
**oxy**	Dissolved oxygen
**PS**	Porous support
**rad**	Radial
**SS**	Strip spacing
**sl**	Stagnant medium layer
**tiss**	Ovarian tissue strip
**tubing**	Inlet/outlet tubing

## Data Availability

The raw data supporting the conclusions of this article will be made available by the authors, without undue reservation.
