# Supplementary figures and images for: Enhanced solute transport and steady mechanical stimulation in a novel dynamic perifusion bioreactor increase the efficiency of the in vitro culture of ovarian cortical tissue strips
Source: Front Bioeng Biotechnol. 2024 Feb 8;12:1310696. doi: 10.3389/fbioe.2024.1310696 (PMC10882273; doi:10.3389/fbioe.2024.1310696)

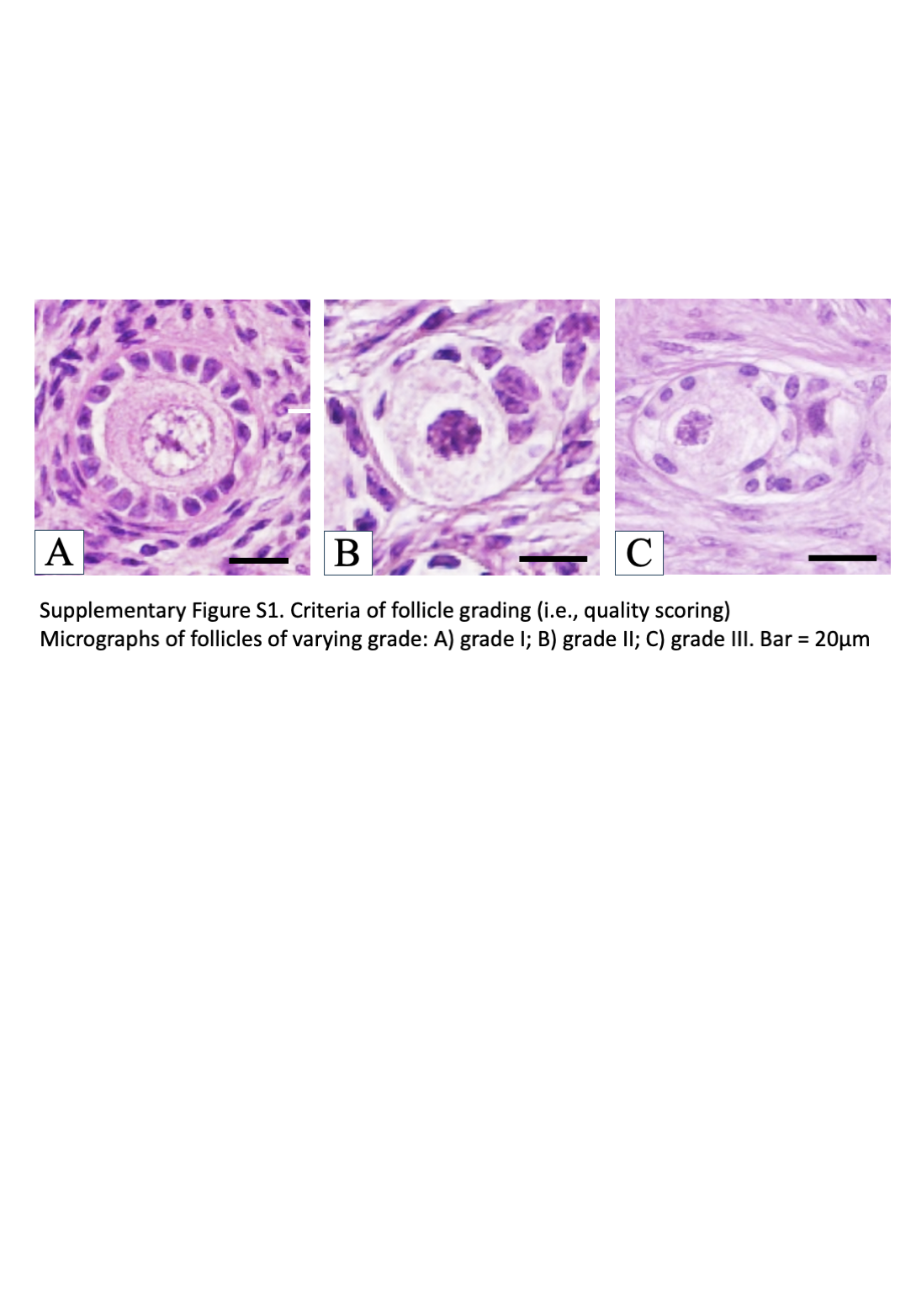

Supplement: Supplementary file 1 [file Image1.TIFF]

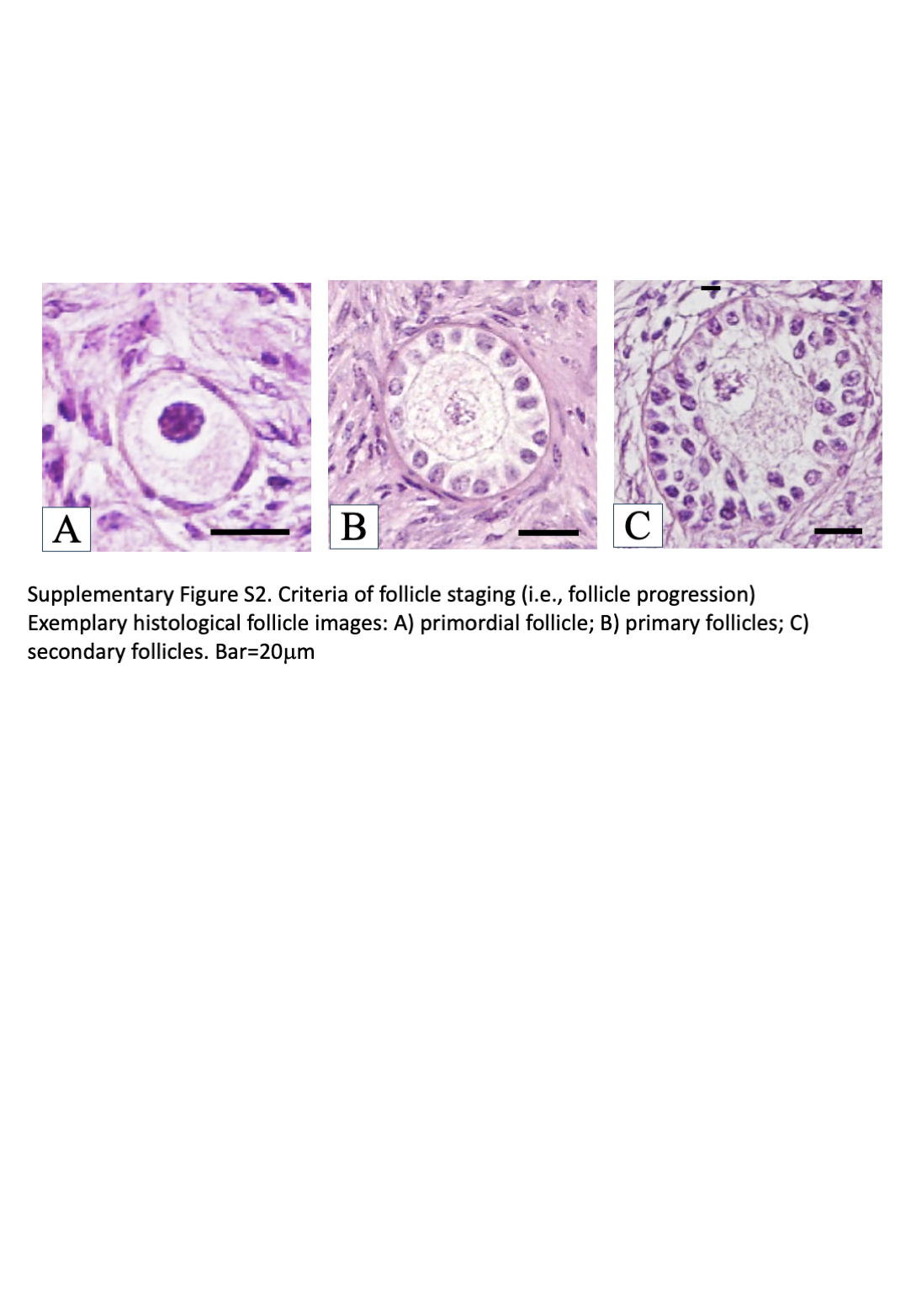

Supplement: Supplementary file 3 [file Image2.TIFF]
