# Supplementary material for: Enhanced solute transport and steady mechanical stimulation in a novel dynamic perifusion bioreactor increase the efficiency of the in vitro culture of ovarian cortical tissue strips
Source: Front Bioeng Biotechnol. 2024 Feb 8;12:1310696. doi: 10.3389/fbioe.2024.1310696 (PMC10882273; doi:10.3389/fbioe.2024.1310696)
Supplement: Supplementary file 2 [file Table1.pdf]

**@1h**	**CTRL**	**µPA**	**PP**	**mDR**	**mPC**	**ABS-L-R**	**LDPE**	**stPA**	**PTR**	**BTR**
**CTRL**		*				*				
**µPA**			*					**		
**PP**										
**mDR**										
**mPC**										
**ABS-L-R**			*					**		
**LDPE**								*		
**stPA**										
**PTR**										
**BTR**										

**@3h**	**CTRL**	**µPA**	**PP**	**mDR**	**mPC**	**ABS-L-R**	**LDPE**	**stPA**	**PTR**	**BTR**
**CTRL**		*				**				
**µPA**			**	*		**		*	**	
**PP**					*	**				
**mDR**										
**mPC**										
**ABS-L-R**				**	**		**	**	**	**
**LDPE**										
**stPA**										
**PTR**										
**BTR**										

@5h	**CTRL**	**µPA**	**PP**	**mDR**	**mPC**	**ABS-L-R**	**LDPE**	**stPA**	**PTR**	**BTR**
**CTRL**		**						**	*	**
**µPA**		**	**		**		**		**	**
**PP**								**	**	**
**mDR**								**	**	**
**mPC**								**	**	**
**ABS-L-R**										
**LDPE**								**	**	**
**stPA**									**	**
**PTR**										
**BTR**										

Supplementary Table 1. Statistical significance of cytocompatibility difference of material candidates for bioreactor construction

Pairwise comparisons of sperm motility progression after 1, 3, and 5 hours incubation in the presence of leachables from various materials according to Fisher's exact test. Significance levels: *p<0.05; **p<0.01.
